# ALDH3A2 negatively orchestrates gastric cancer progression through a synergistic induction of ferroptosis and ferroptosis-driven macrophage reprogramming

**DOI:** 10.1038/s41419-025-08364-8

**Published:** 2025-12-24

**Authors:** Yuanyuan Ren, Yue Cui, Zhen Wang, Yizhi Luo, Junchang Jin, Yiyi Yuan, Xuan Li, Yaning Zhang, Nan Cao, Xiaofang Li, Yi Yu, Yuyan Xiong

**Affiliations:** 1 Key Laboratory of Resource Biology and Biotechnology in Western China, Ministry of Education, College of Life Sciences, Northwest University, Xi’an, Shaanxi PR China; 2 Xi’an Key Laboratory of Cardiovascular and Cerebrovascular Diseases, Xi’an No. 3 Hospital, The Affiliated Hospital of Northwest University, Xi’an, Shaanxi PR China; 3 School of Medicine, Northwest University, Xi’an, Shaanxi PR China; 4 Department of Gastroenterology, Xi’an No. 3 Hospital, The Affiliated Hospital of Northwest University, Xi’an, Shaanxi PR China

**Keywords:** Gastric cancer, Cell death

## Abstract

Gastric cancer (GC) is a prevalent gastrointestinal malignancy in which ferroptosis, mitochondrial dysfunction, and macrophage reprogramming remarkably contribute to disease progression. However, the molecular interplay among these processes in contributing to GC remains poorly understood. In this study, we identified ferroptosis- and mitochondrial dysfunction-related genes (FMDRGs) implicated in GC through bioinformatics analyses. Among them, aldehyde dehydrogenase 3 family member A2 (ALDH3A2) was identified as a key FMDRG significantly downregulated in GC tissues and cell lines. Functional assays revealed that ALDH3A2 overexpression in GC cell lines suppressed proliferation, migration, and invasion while enhancing ferroptosis, effects that were reversed by GPX4 overexpression. ALDH3A2 also impaired the mitochondrial unfolded protein response (UPR^mt^) and induced mitochondrial dysfunction. Restoration of UPR^mt^ ameliorated ALDH3A2-induced mitochondrial dysfunction and ferroptosis. Mechanistically, ALDH3A2 impaired UPR^mt^ by downregulating SLC47A1 through blockade of NRF2 nuclear translocation, leading to mitochondrial dysfunction, GPX4 downregulation, lipid peroxidation, and subsequent ferroptosis. Synergistically, ALDH3A2-induced ferroptosis promoted IL-6 release, which drove macrophage polarization toward the M1 phenotype with elevated IL-1β production. This macrophage reprogramming, in turn, inhibited GC cell progression by downregulating PD-L1 expression. Therapeutically, both genistein treatment and ALDH3A2 overexpression significantly attenuated GC progression in vitro and in vivo. These findings elucidate ALDH3A2 as a dual regulator of tumor-intrinsic ferroptosis and tumor-extrinsic immune remodeling in contributing to GC pathogenesis, highlighting its potential as a promising therapeutic target in GC.

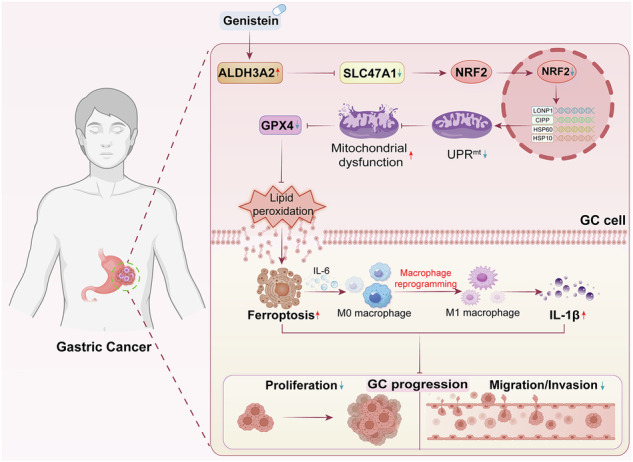

## Introduction

Gastric cancer (GC) ranks as the fifth most prevalent cancer and the third leading cause of cancer-related mortality worldwide [1]. Despite significant advancements in diagnosis and treatment, the prognosis for gastric cancer remains poor, particularly in its advanced stages [2]. This poor outcome is largely attributed to the incomplete understanding of the molecular mechanisms driving tumorigenesis and metastasis, which also hinders the development of effective therapeutic strategies. As such, elucidating the intrinsic molecular mechanisms of GC is essential for improving GC patient therapy outcomes.

Ferroptosis, an iron-dependent form of cell death characterized by the accumulation of iron and excessive lipid peroxidation [3], can be initiated through two principal pathways. The exogenous pathway involves inhibition of the glutamate/cystine antiporter (system Xc^-^) and activation of iron transporters such as serotransferrin and lactotransferrin, whereas the endogenous pathway primarily results from impaired biosynthesis or activity of the intracellular antioxidant enzyme glutathione peroxidase 4 (GPX4) [4]. Increasing evidence suggests that ferroptosis exerts tumor-suppressive effects by restricting cell proliferation, invasion, and metastasis. In GC, multiple studies have shown that the induction of ferroptosis effectively suppresses tumor growth and improves patient prognosis [5–7].

Mitochondria are central hubs for energy production and cellular metabolism [8]. Mitochondrial dysfunction, arising from mtDNA mutations, defective tricarboxylic acid (TCA) cycle enzymes, electron transport chain leakage, oxidative stress, or dysregulated oncogenic and tumor-suppressor signaling, profoundly alters metabolic pathways, disrupts redox homeostasis, and promotes therapeutic resistance, thereby facilitating tumorigenesis [9]. Importantly, ferroptosis is tightly linked to mitochondrial activity, including mitochondrial damage and dysfunction enhances oxidative stress and lipid peroxidation, ultimately driving ferroptotic cell death [3]. Nonetheless, the specific molecular mechanisms of ferroptosis linking to mitochondrial function in contributing to GC pathogenesis still remain poorly understood.

In this study, comprehensive bioinformatics analyses were conducted to identify key FMDRGs implicated in the pathogenesis of GC. Next, a series of in vitro and in vivo experiments were performed to investigate the underlying mechanisms by which the identified FMDRGs contribute to GC progression. Among them, ALDH3A2 was identified as a key FMDRG that suppresses GC progression through a synergistic mechanism involving the induction of ferroptosis and promotion of ferroptosis-driven macrophage reprogramming.

## Materials and methods

### Acquisition of ferroptosis-related genes

The public FerrDb database (http://www.zhounan.org/ferrdb), containing ferroptosis-related genes (FRGs) that promote, inhibit, or mark ferroptosis, was also queried. After removing duplicates, 910 unique FRGs were identified for further analysis [10]. Mitochondrial dysfunction-related genes were sourced from the MitoProteome database (http://mitoproteome.org//), which offers extensive data on human genes. The search term “mitochondrial dysfunction” was used, and only genes with relevance scores above 1 were selected.

### Collection of datasets

RNA-seq data and clinical characteristics for the TCGA STAD cohort were obtained from the TCGA website (https://portal.gdc.cancer.gov/) for training purposes. Genome sequencing in these patients was conducted prior to treatment, as TCGA primarily focuses on untreated primary cancers [11]. Participants lacking detailed expression and clinical data or with a follow-up duration of zero days were excluded [12]. Based on these criteria, a total of 412 STAD samples and 36 normal samples were selected for the training cohort.

### Differential expression analysis

Differential expression analysis between TCGA STAD and normal gastric tissue was performed using the edgeR package (https://bioconductor.org/packages/release/bioc/html/edgeR.html) and visualized through volcano plots. Differentially expressed genes (DEGs) were identified based on a significance threshold of p < 0.05 and |fold change (FC)| >1 [13]. The intersection of DEGs with ferroptosis- and mitochondrial dysfunction-related genes, as shown in a Venn diagram, was selected for further analysis.

### Feature selection and prognostic model construction

To identify key FMDRGs associated with patient prognosis, we employed a multi-step feature selection strategy integrating Least Absolute Shrinkage and Selection Operator (LASSO)-Cox regression, the Boruta algorithm, and Random Forest analysis. LASSO-Cox regression was performed using the *glmnet* R package [14], which penalizes regression coefficients while performing variable selection, thereby retaining prognostic genes with non-zero coefficients linked to overall survival. The Boruta algorithm, implemented with the *Boruta* R package [15], served as a Random Forest–based wrapper that iteratively compares the importance of original features against permuted “shadow” features, preserving only truly relevant variables. To further assess gene importance in survival prediction, we employed Random Survival Forests using the *randomForestSRC* R package [16], an ensemble tree-based approach tailored for right-censored survival data. Genes consistently identified across all three methods were considered robust predictors and were used to construct the prognostic model.

### Reagents

Reagents were purchased or obtained from the following sources: rabbit anti-ALDH3A2 antibody (A19899, ABclonal, China), rabbit anti-SLC47A1 antibody (20898-1-AP, Proteintech Wuhan, China), rabbit anti-NRF2 antibody (16396-1-AP, Proteintech Wuhan, China), rabbit anti-LONP1 antibody (A4293, ABclonal, China), rabbit anti-GPX4 antibody (A1933, ABclonal, China), mouse anti-HSP60 antibody (66041-1-Ig, Proteintech Wuhan, China), mouse anti-PD-L1 antibody (66248-1-Ig, Proteintech Wuhan, China), mouse anti-Beta Actin antibody (66009-1-Ig, Proteintech Wuhan, China), mouse anti-β-Tubulin antibody (AC021-1-Ig, ABclonal, China), rabbit anti-PCNA antibody (A13336, ABclonal, China), and goat anti-rabbit IgG (H + L) secondary antibody Alexa Fluor® 488 conjugate (ABclonal, China). Plasmids (psPAX2, pMD2.G) used in this experiment were purchased from Vector Builder (China). Cell Counting Kit-8 assay was purchased from Beyotime (Cat No.C0038, Beyotime, China). Dimethyl Fumarate was purchased from Solarbio (YS144741, Solarbio, China), Bezafibrate, Bicalutamide, Genistein, and Rosiglitazone were purchased from Aladdin (B129520, B118360, G106673, R128083, Aladdin, China), Arsenic trioxide was purchased from Sigma (Cat#202673, Sigma, USA), Oligomycin was purchased from GLPBIO (GC16533, GLPBIO, USA), and N-Acetylcysteine (NAC) was purchased from MCE (HY-B0215, MCE, USA). ROS fluorescence probe DCFH-DA was obtained from Beyotime (S0033S, Beyotime, China), and Mitochondrial Membrane Potential Fluorescent Probe JC-1 was purchased from Solarbio (M8650, Solarbio, China). All cell culture media and materials were obtained from Gibco (USA).

### Cell cultures

Human normal gastric mucosal epithelial cells GES-1, gastric cancer cell lines MGC803, HGC27, SGC7901, MKN45, and human embryonic kidney cells (293 T) used in this study were all sourced from Procell (Wuhan, China). These cells were cultured in DMEM medium (Sigma, USA) supplemented with 10% FBS, 100 U/mL penicillin, and 100 µg/mL streptomycin, and maintained in a humidified chamber with 5% CO₂ at 37 °C. All cell lines were authenticated by short tandem repeat (STR) analysis and confirmed to be free of mycoplasma contamination.

### Lentivirus generation and transduction

HEK-293T cells were co-transfected with the packaging plasmid (psPAX2), envelope plasmid (pMD2.G), and the PLKO.1-TRC plasmid with targeted shRNA sequences for knockdown or the PLV-EF1a plasmid with ALDH3A2 gene sequences for overexpression using Lipo6000^TM^ Transfection Reagent to produce the corresponding lentivirus. After culturing the transfected HEK-293T cells for 24 h and 48 h, the supernatant was removed and centrifuged for 5 min at 1000 rpm to extract virus particles. Subsequently, lentivirus-infected cells were screened for cells expressing the relevant antibiotic resistance gene in a growth medium supplemented with 2 µg/mL puromycin.

For overexpression of ALDH3A2, the ALDH3A2 gene underwent amplification from the template plasmid PLV-EF1a-ALDH3A2 via PCR, employing the forward primer: 5’- GCCACCACGCGTCGACACCATGGAGCTCGAAGTCC -3’, and the reverse primer sequence: 5’-AGCTGGGTGCTCTAGTCATCTCTGCTTACTGGACCAACG-3’. To construct the SLC47A1 overexpression vector, the SLC47A1 gene was amplified from genomic DNA extracted from the gastric cancer cell (MGC803) using PCR. The forward primer sequence was 5’- GCCACCACGCGTCGACACCATGGAAGCTCCTGAGGAG -3’, and the reverse primer sequence was 5’- AGCTGGGTGCTCTAGTCACTGAATTCTGACATAGAATCTC -3’. To interfere with IL-6 expression, short hairpin RNA (shRNA) oligos of IL-6 targeting IL-6 were cloned into pLKO.1-TRC. The boldface sequences below represent the targeting sequences for hIL-6-shRNA (only the sense strand is shown):

pLKO.1-hIL-6-F:

5’-CCGG**CAGAACGAATTGACAAACAAA**CTCGAGTTTGTTTGTCAATTCGTTCTGTTTTTG-3’

To interfere with IL-6R expression, short hairpin RNA (shRNA) oligos of IL-6R targeting IL-6R were cloned into pLKO.1-TRC. The boldface sequences below represent the targeting sequences for hIL-6R-shRNA (only the sense strand is shown):

pLKO.1-hIL-6R-F:

5’-CCGG**GCAGGCACTTAATACTAATAA**CTCGAGTTATTAGTAGTAAGTGCCTGCTTTTTG-3’

To interfere with IL-1β expression, short hairpin RNA (shRNA) oligos of IL-1β targeting IL-1β were cloned into pLKO.1-TRC. The boldface sequences below represent the targeting sequences for hIL-1β-shRNA (only the sense strand is shown):

pLKO.1-hIL-1β-F:

5’-CCGG**GCGATTTGTCTTCAACAAGAT**CTCGAGATCTTGTTGAAGACAAATCGCTTTTTG-3’

### Immunoblotting

Cell extracts were prepared by lysing cells in RIPA buffer, and protein concentrations were determined using the Pierce BCA Protein Assay Kit. Subsequently, 20 μg of denatured protein extracts were separated via electrophoresis on a 10–12.5% SDS-polyacrylamide gel and transferred to polyvinylidene difluoride membranes (BioRad Laboratories, Hertfordshire, UK). After blocking with 5% skimmed milk, membranes were incubated with primary antibodies at room temperature for 2 hours or overnight at 4 °C. Following this, membranes were washed and incubated with secondary antibodies for 1.5 hours at room temperature. Immunoreactive proteins were visualized using the Tanon 5200 Luminescent Imaging System with an ECL Enhanced Kit (ABclonal, Wuhan, China) and quantified by densitometry with ImageJ software. β-ACTIN was used as a loading control.

### RNA extraction and quantitative real-time polymerase chain reaction

Total RNA was extracted using Trizol (ThermoFisher, Waltham, MA, USA) following the manufacturer’s instructions. For cDNA synthesis, 1 μg of total RNA from each cell line was reverse transcribed using the TransScript® One-Step gDNA Removal and cDNA Synthesis SuperMix (Beijing Quanshijin Biotechnology Co., Ltd., Beijing, China). Quantitative real-time PCR (qRT-PCR) was performed using PerfectStart Green qPCR SuperMix on a Bio-Rad CFX96 Real-Time PCR system (Bio-Rad, USA) with the following program: 94 °C for 30 seconds, followed by 45 cycles at 94 °C for 5 seconds and 60 °C for 30 seconds. Relative gene expression levels were calculated using the 2-ΔΔCt method, with GAPDH as an internal control. The primer sequences used in this experiment were listed as follows:

List of primers used for qRT-PCR (h: human; m: mouse).

**Table:** 

Target gene	Forward	Reverse
hALDH3A2	ACTGATAGGAGCCATCGCTGCA	GCTCCGTGGTTTCCTCAACACC
hSLC47A1	TGCTGTAGCCTTCAGTGTCCTG	GCTTCAAAGAGGTGGGAAACAGC
hLONP1	TGGAGGAAGACCACTACGGCAT	GCCATAGAAGCAGAGGATCTTGC
hHSP60	TGCCAATGCTCACCGTAAGCCT	AGCCTTGACTGCCACAACCTGA
hPPP3CB	CTTGGCGATTATGTGGACAGAGG	GGTGTCTGCATTCATGGTTGCC
hPDL1	TGCCGACTACAAGCGAATTACTG	CTGCTTGTCCAGATGACTTCGG
hIL-6	AGACAGCCACTCACCTCTTCAG	TTCTGCCAGTGCCTCTTTGCTG
hIL-1α	TGTATGTGACTGCCCAAGATGAAG	AGAGGAGGTTGGTCTCACTACC
hIL-1β	CCACAGACCTTCCAGGAGAATG	GTGCAGTTCAGTGATCGTACAGG
hAKR1B10	GAGGACCTGTTCATCGTCAGCA	CGTCCAGATAGCTCAGCTTCAG
hCA2	GTGACCTGGATTGTGCTCAAGG	GTTGTCCACCATCAGTTCTTCGG
hMUC5AC	CCACTGGTTCTATGGCAACACC	GCCGAAGTCCAGGCTGTGCG
hSFRP2	CTCCAAAGGTATGTGAAGCCTGC	CCAGGATGATTTTGGTATCTCGG
hAPOD	CAACTACTCACTAATGGAAAACGG	CTTGGCAGGCTCTGTGAGGTTA
hHSPB6	GCCACTTTTCGGTGCTGCTAGA	GCGCGACGAATCCGTGCTCAT
mALDH3A2	GATCTTGGCTGAACTCCTCCCT	GAGAATGTGGTCAAACCGCTGC
mSLC47A1	GCTCTTTGCTGTGACCTTCTGC	GACCTTCAAAGAGGTGGGACAC
mLONP1	CCAAGCATGTGATGGACGTGGT	GTCCAAGTTCTCATCACTCTGCC
mHSP60	TGCTCATCGGAAGCCATTGGTC	TTGACTGCCACAACCTGAAGACC
mGAPDH	CATCACTGCCACCCAGAAGACTG	ATGCCAGTGAGCTTCCCGTTCAG

### Colony formation assay

Approximately 500 cells per group were seeded in six-well plates in triplicate and incubated at 37 °C with 5% CO₂ for about 2 weeks, until visible colony formation was observed under a microscope. Colonies were then fixed with 4% paraformaldehyde for 20 minutes and washed twice with PBS. Afterward, cells were stained with 1 mL of 0.2% crystal violet solution (Sigma, St. Louis, MO, USA) for 10 minutes, followed by three washes with PBS. The plates were subsequently air-dried, imaged, and colony numbers were quantified using ImageJ software.

### Cell counting kit 8 (CCK8) assay

Cell proliferation was assessed in this study using the Cell Counting Kit-8 (CCK8) assay. Cells from each group were seeded in 96-well plates at a density of 5 × 10³ cells per well and incubated at 37 °C with 5% CO₂ for 24, 48, 72, and 96 hours. After each time point, 10 μL of CCK8 solution was added to each well, followed by a 2-hour incubation in the dark. The absorbance at 450 nm was then measured using a microplate reader (Bio-Rad Laboratories, Hercules, CA, USA).

### Wound healing assay

A scratch wound assay was performed to assess the migration ability of GC cells in vitro. Horizontal reference lines were drawn on the back of a 6-well plate, and cells were seeded into the wells for culture. Once the cells reached approximately 80% confluence, a sterile 200 µL pipette tip was used to create perpendicular scratches across the cell monolayer, after which the cells were washed with PBS. The medium was then replaced with DMEM containing 0.2% FBS, and cells were incubated at 37 °C with 5% CO₂ for 24 hours. Scratch images were captured at 0 and 24 hours using a light microscope (Olympus Corp, Tokyo, Japan), and the scratch area was quantified with ImageJ.

### Transwell assay

Add 100 μL of diluted Matrigel to the upper chamber of the transwell insert and incubate in a 5% CO₂ incubator at 37 °C for 2-4 hours to allow Matrigel to solidify. Next, add 200 μL of a cell suspension containing 5 × 10⁴ cells in serum-free medium to the upper chamber, while placing 500 μL of complete medium with 10% FBS in the lower chamber. Incubate the transwell setup in a 5% CO₂ incubator at 37 °C for 24 hours. After incubation, wash the transwell chambers three times with PBS, remove non-invading cells from the upper chamber using a cotton swab, then fix with 4% paraformaldehyde for 20 minutes. Wash three times with PBS, then stain with 0.25% crystal violet for 30 minutes. Afterward, wash three times with PBS, allow to dry, and photograph.

### Subcellular fractionation

To perform subcellular fractionation, cells were harvested from 10 cm plates and resuspended in 500 μL of fractionation buffer containing 20 mM HEPES (pH 7.4), 10 mM KCl, 2 mM MgCl₂, 1 mM EDTA, 1 mM EGTA, 1 mM DTT, and a protease inhibitor cocktail (PI Cocktail III). The suspension was incubated on ice for 15 minutes, followed by cell lysis achieved by repeatedly passing the suspension through a 27-gauge needle with a 1 mL syringe. The lysate was incubated on ice for an additional 20 minutes. Samples were then centrifuged at 3000 rpm for 5 minutes, and the supernatant, containing cytoplasm, membrane, and mitochondria, was carefully transferred to a new tube and kept on ice. The nuclear pellets were washed with 500 μL of fractionation buffer, dispersed using pipettes, and passed through a 25-gauge needle 10 times. After a second centrifugation at 3,000 rpm for 10 minutes, the nuclear pellets were resuspended in TBS with 0.1% SDS and briefly sonicated (3 seconds on ice at a power setting of 2-continuous) to shear genomic DNA and homogenize the lysate. For cytoplasmic preparation, the supernatant containing cytoplasm, membrane, and mitochondria was centrifuged at 8000 rpm for 5 minutes. The final supernatant, containing cytoplasm and membrane, was transferred to a fresh tube and stored on ice or at -80 °C for future use.

### Immunofluorescence staining

Glass coverslips were placed in six-well plates, and cells were seeded onto these coverslips. Before staining, cells were fixed with 4% paraformaldehyde for 15 minutes at room temperature and washed twice with cold PBS. The cells were then permeabilized with 0.2% Triton X-100 and blocked in PBS containing 1% BSA for 60 minutes at room temperature. After three washes with cold PBS, cells were incubated with the primary antibody at 4 °C overnight, followed by incubation with an Alexa Fluor-labeled secondary antibody for 2 hours at room temperature. Coverslips were mounted, and images were captured using a Leica TCS SP5 confocal laser microscope with a 40× objective lens. Representative images were taken at consistent exposure and magnification for all samples.

### Reactive oxygen species assay

Cells were seeded at a density of 10,000 cells/cm² and allowed to adhere. After treatment, cells were washed twice with PBS and incubated with 10 μM DCFH-DA and 5 μM DAF-FM DA in serum-free medium for 20 minutes at 37 °C in the dark. Following incubation, cells were washed three times with PBS to remove any unbound dye. Reactive oxygen species (ROS) generation was then visualized using fluorescence microscopy (Olympus, Tokyo, Japan).

### Mitochondrial membrane potential (MMP, ΔΨm) measurement

Cells were seeded at a density of 10,000 cells/cm² and allowed to adhere. JC-1 dye was then applied to assess mitochondrial membrane potential (ΔΨm). A high ΔΨm facilitates JC-1 aggregation in the mitochondrial matrix, producing red fluorescence, whereas a low ΔΨm results in JC-1 monomers with green fluorescence. After staining, cells were qualitatively observed using fluorescence microscopy (Olympus, Tokyo, Japan) and quantitatively assessed by flow cytometry to measure mitochondrial membrane potential (MMP).

### Malondialdehyde level detection

Cellular malondialdehyde (MDA) levels, a key end product of lipid peroxidation, were measured using an MDA content detection kit (Solarbio, Beijing, China). Briefly, according to the manufacturer’s protocol, 5 × 10⁶ cells were lysed with 1 mL of extraction solution and centrifuged at 4 °C for 10 minutes at 8000 g, with the supernatant collected and kept on ice for analysis. Next, 200 μL of supernatant was combined with 600 μL of MDA working solution, mixed thoroughly, and incubated at 100 °C for 60 minutes. After cooling, the mixture was centrifuged at 10,000 g at room temperature for 10 minutes. The supernatant was then transferred to a 96-well plate, and absorbances at 532 nm and 600 nm were recorded using a microplate reader (Bio-Rad Laboratories, Hercules, CA, USA).

### Flow cytometry analysis

The treated cells were digested and collected with trypsin, washed with PBS solution containing 0.5% BSA, and then centrifuged at 400×g for 3 minutes at 4 °C. The supernatant was removed. Next, resuspend the cells in PBS containing 0.5% BSA and evenly distribute them into 96-well plates at 50 μL per well (1 × 10^6^ cells per well). Then, add the PE anti-human CD86 antibody (A26125, ABclonal, China) and APC anti-human CD11b antibody (A26893, ABclonal, China), 50 μL per well, and the mixture was incubated at room temperature in the dark for 20 minutes. After incubation, cells were washed with PBS, detected using flow cytometry (FACS Calibur, BD, USA), and the data were analyzed using FlowJo V.10.8.1.

### Animal studies

Male BALB/c nude mice (4-5 weeks old) used in this study were obtained from Jiangsu JiCui YaoKang Biotechnology Co., LTD. (Jiangsu, China). The mice were housed under specific pathogen-free conditions with a 12-hour light/dark cycle and provided with autoclaved laboratory rodent diet. Tumor development studies assessing the impact of ALDH3A2 were initiated when the mice reached 6 weeks of age. Nude mice were randomly assigned to two groups (n = 5 per group). Each mouse received a subcutaneous injection of 200 μL of cell suspension containing either EF1a-ALDH3A2 or a negative control stably transfected MGC803 cell line (2.5 × 10⁷ cells/mL in PBS) into the right armpit. To examine the effects of genistein on tumor development, all experimental mice first received a subcutaneous injection of 200 μL MGC803 cell suspension (2.5 × 10⁷ cells/mL) into the right armpit. After one week, mice were randomly divided into three groups (n = 5 per group). Two groups were intraperitoneally (i.p.) injected with genistein (dissolved in DMSO, diluted in PBS) at daily doses of 50 mg/kg or 100 mg/kg body weight for four consecutive weeks, while the control group received only the vehicle. Body weight was recorded daily. Tumor volume (V = length × width²/2) was measured weekly with a caliper throughout the experiment. At the end of the treatment, mice were sacrificed, and subcutaneous tumor tissues were collected for qRT-PCR and western blot analysis. All animal experiments were approved by the Animal Ethics Review Committee of Northwestern University (Approval No. NWU-AWC-20221105M) and conducted in accordance with the guidelines outlined in EU Directive 2010/63/EU.

### Statistical analysis

All data are presented as mean ± SEM from 4 to 8 independent experiments, with n (number of experimental subjects or animals) specified in the figure legends. Statistical significance between two groups was determined by Student’s t-test, while comparisons across multiple groups were assessed by one-way analysis of variance (ANOVA) followed by Bonferroni’s post hoc test. Statistically significant differences are indicated by asterisks (*p < 0.05; **p < 0.01; ***p < 0.001).

## Results

### ALDH3A2 was identified as a potential key ferroptosis- and mitochondrial dysfunction-related gene in contributing to GC progression

**Fig. 1:**
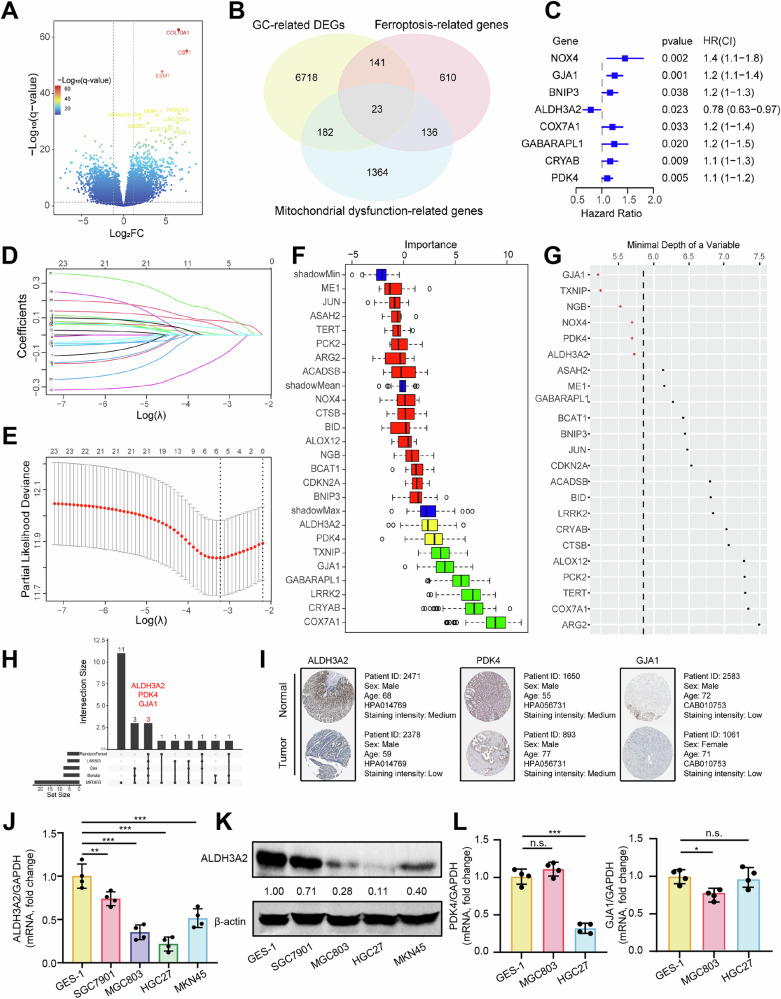
ALDH3A2 is identified as a key ferroptosis- and mitochondrial dysfunction-related gene implicated in gastric cancer. **A** Volcano plot of differentially expressed genes between gastric cancer and adjacent non-tumor tissues. **B** Venn diagram showing the overlap of GC-related differentially expressed genes (DEGs) with ferroptosis-related genes (FerrDb) and mitochondrial dysfunction-related genes (MitoProteome). Prognostic gene screening based on overall survival using Univariate Cox regression (**C**), LASSO Cox analysis (**D**, **E**), Boruta (**F**), and Random Forest (**G**). **H** Upset plot showing ALDH3A2, PDK4, and GJA1 as common candidate genes identified across all machine learning methods. **I** Immunohistochemistry images of ALDH3A2, PDK4, and GJA1 proteins from the Human Protein Atlas. **J** Relative mRNA expression of ALDH3A2 in GES-1, SGC7901, MGC803, HGC27, and MKN45 cells assessed by qRT-PCR (n = 4). **K** Protein expression of ALDH3A2 in GES-1, SGC7901, MGC803, HGC27, and MKN45 cells were assessed by immunoblotting (n = 4). **L** Relative mRNA expression of PDK4 and GJA1 in GES-1, HGC27, and MGC803 cells assessed by qRT-PCR (n = 4). Statistical significance was determined by an unpaired Student’s t-test. Data are presented as mean ± SEM. *p < 0.05, **p < 0.01, ***p < 0.001; n.s., not significant.

To identify the key ferroptosis- and mitochondrial dysfunction-related genes (FMDRGs) involved in the pathogenesis of GC, we analyzed 448 samples from The Cancer Genome Atlas-Stomach Adenocarcinoma (TCGA-STAD), including 36 adjacent non-tumor tissues and 412 tumor tissues. The characteristic information of the patients is detailed in Supplementary Table 1. Differential expression analysis of 33 paired tumor and adjacent non-tumor samples revealed 7064 GC-differentially expressed genes (GC-DEGs) ( | log2FC | > 1, p < 0.05), with 3423 upregulated and 3,641 downregulated in GC tissues (Fig. 1A). Next, we sourced 910 ferroptosis-regulated genes (FRGs) from FerrDb and 1,705 mitochondrial dysfunction-related genes (MDRGs) from MitoProteome. A Venn diagram analysis of FRGs, MDRGs, and GC-DEGs identified 23 FMDRGs potentially linking ferroptosis and mitochondrial dysfunction in GC (Fig. 1B). To assess the prognostic relevance of 23 FMDRGs, we analyzed their associations with overall survival (OS) in GC patients from the TCGA-STAD cohort. Univariate Cox analysis identified 8 FMDRGs significantly correlated with the overall survival (OS) of STAD patients (Fig. 1C). Additionally, LASSO Cox, Boruta, and Random Forest analyses identified 6, 8, and 6 FMDRGs, respectively, contributing to the survival of STAD patients (Fig. 1D–G). Notably, three key FMDRGs (ALDH3A2, PDK4, and GJA1) were consistently identified across all four algorithms (Fig. 1H).

To further validate the impact of these three FMDRGs on patient survival, we constructed a prognostic model using multivariate Cox regression. The global Schoenfeld test (p = 0.8047) and individual test p-values for each variable (PDK4: p = 0.7927; ALDH3A2: p = 0.4228; GJA1: p = 0.6397) confirmed that the proportional hazards assumption was satisfied, with no systematic trends or significant deviations over time (Fig. S1A–C). The coefficients for PDK4, ALDH3A2, and GJA1 were 0.06788057, 0.29799055, and 0.17942534, respectively (Fig. S2A). We calculated each patient’s risk score using the formula:${RiskScore}=\sum {{\rm{coef}}}_{i}* {{\rm{geneExpr}}}_{i}$, and categorized patients into high- and low-risk groups based on the median RiskScore (0.05339532) (Fig. S2B). Our analysis demonstrated a strong concordance between the risk score derived from the three-gene model and actual overall survival (Fig. S2C). Notably, patients in the low-risk group exhibited significantly longer overall survival compared to those in the high-risk group (p = 0.00014) (Fig. S2D). Moreover, we performed prognostic analyses correlating the risk score with other common clinical features. Both univariate (p < 0.001, HR 2.8 [95%CI 1.6-5.1]) (Fig. S2E) and multivariate Cox analyses (p < 0.001, HR 2.7 [95%CI 1.7-4.3]) (Fig. S2F) indicated that our risk score is a significant predictor of overall survival in STAD patients. To validate the protein expression of these three key FMDRGs in GC tissues, we analyzed immunohistochemical (IHC) data from the Human Protein Atlas (HPA) database. Compared to normal tissues, the staining intensity of ALDH3A2 was remarkably reduced in GC tissues, while PDK4 and GJA1 showed no notable changes (Fig. 1I). Additionally, in vitro analyses of GC cell lines (MGC803, HGC27, SGC7901, and MKN45) revealed significantly reduced ALDH3A2 mRNA and protein levels compared with the normal gastric epithelial cell line GES-1 (Fig. 1J, K), suggesting a potential tumor-suppressive role of ALDH3A2 in contributing to GC progression. Given the lower ALDH3A2 expression, MGC803 and HGC27 were selected for subsequent in vitro studies. Of note, qRT-PCR analysis showed no significant changes in PDK4 and GJA1 mRNA expression in either MGC803 or HGC27 compared with GES-1 cells (Fig. 1L). Furthermore, we performed transcriptomic stratification of the TCGA cohort by dividing patients into ALDH3A2-high and ALDH3A2-low groups based on the median expression value. DEGs between these two groups were identified using the “limma” package with an adjusted p-value < 0.05 and |log2FC | > 2, and the resulting volcano plot highlighted a distinct set of significantly altered genes (Fig. S2G). Among them, the top three upregulated genes (MUC5AC, CA2, and AKR1B10) and the top three downregulated genes (SFRP2, APOD, and HSPB6) were selected for validation. qRT-PCR analyses in ALDH3A2-overexpressing GC cell lines confirmed expression patterns consistent with the TCGA data (Fig. S2H, I). These findings further confirm strong concordance between in silico analyses and experimental validation and further support the functional involvement of ALDH3A2 in GC progression.

### Overexpression of ALDH3A2 suppresses GC cell progression along with enhanced ferroptosis

**Fig. 2:**
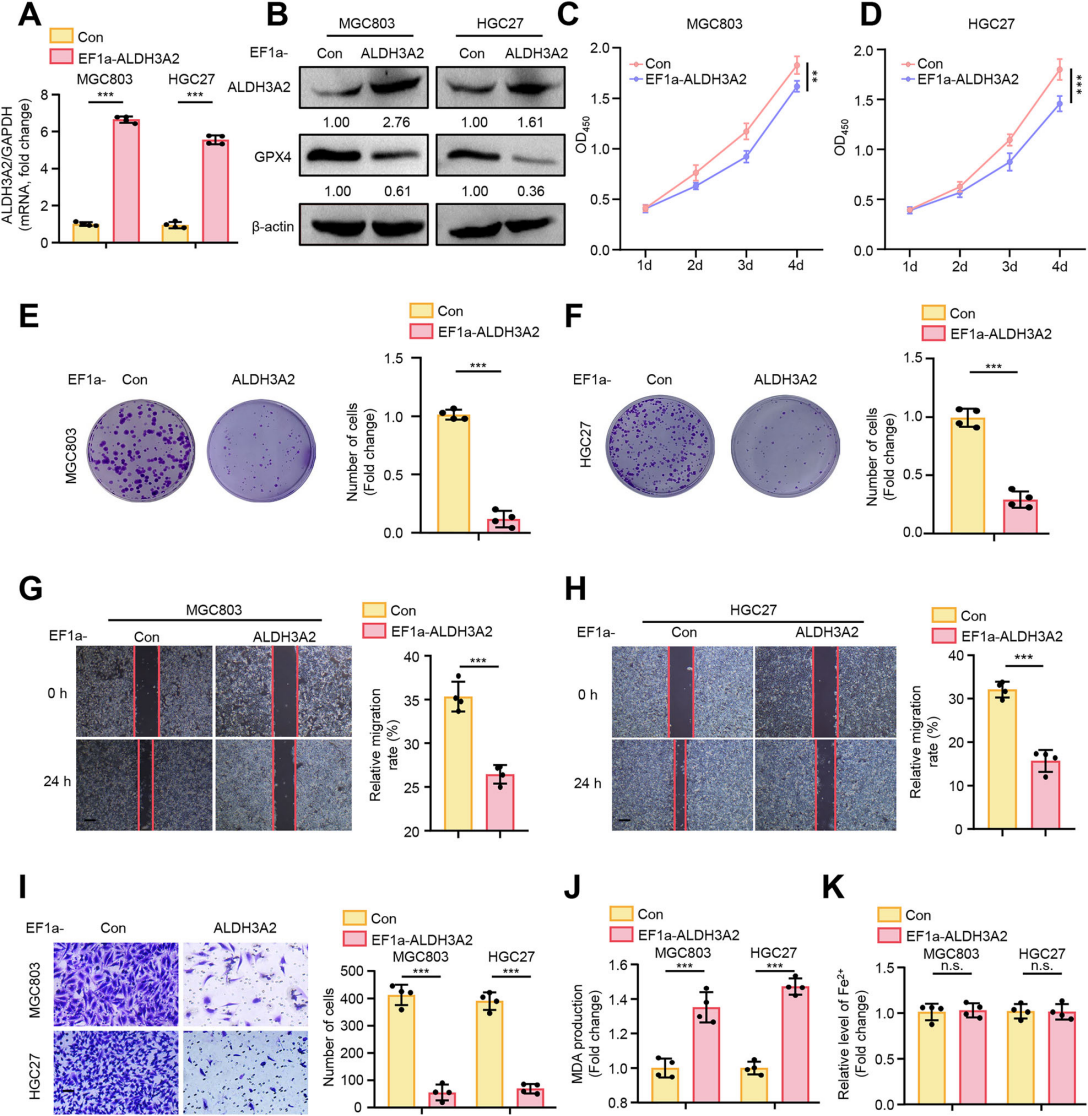
ALDH3A2 overexpression suppresses the aggressive progression of GC cells along with induction of ferroptosis. MGC803 and HGC27 cells were transduced with either PLV-EF1a empty lentivirus as a control (Con) or PLV-EF1a-ALDH3A2 to overexpress ALDH3A2. **A** qRT-PCR analysis of ALDH3A2 expression (n = 4). **B** Immunoblotting analysis of ALDH3A2 and GPX4 protein levels; quantification relative to β-actin is shown below the bands (n = 4). **C**, **D** CCK-8 assays (n = 8) and **E**, **F** colony formation assays (n = 4) assessing cell proliferation. **G**, **H** Wound-healing assays; images were captured at 0 h and 24 h post-scratching, with quantification of wound closure shown in bar graphs (n = 4). **I** Transwell invasion assays (n = 4). **J** Malondialdehyde (MDA) content and **K** intracellular Fe²⁺ levels following ALDH3A2 overexpression, with quantification shown in bar graphs (n = 4). Data are presented as mean ± SEM. Statistical significance was determined using an unpaired Student’s t-test. **p < 0.01, ***p < 0.001, n.s., not significant. Scale bar = 0.1 mm.

To examine the suppressive role of ALDH3A2 in modulating the malignancy of GC cell progression, we initially overexpressed ALDH3A2 in MGC803 and HGC27 GC cell lines, as confirmed by qPCR and immunoblotting analysis (Fig. 2A, B). CCK8 and colony formation assays indicated that ALDH3A2 overexpression dramatically inhibited cell growth and colony formation in both GC cell lines (Fig. 2C–F). To assess the impact of ALDH3A2 on GC metastasis, wound healing, and transwell invasion assays were performed. Notably, ALDH3A2 overexpression markedly suppressed cell migration (Fig. 2G, H) and invasion (Fig. 2I). Immunoblotting analysis indicated that ALDH3A2 overexpression in GC cells substantially reduced GPX4 expression (Fig. 2B), while promoting lipid peroxidation, evidenced by elevated MDA levels (Fig. 2J). However, no significant changes were observed in intracellular Fe²⁺ levels compared to the control group (Fig. 2K). These results prompt us to infer that ALDH3A2 might suppress GC cell progression and promote ferroptosis through a GPX4-dependent manner.

### ALDH3A2 exhibits suppressive effects on the progression of GC cells via inducing ferroptosis in a GPX4-dependent manner

**Fig. 3:**
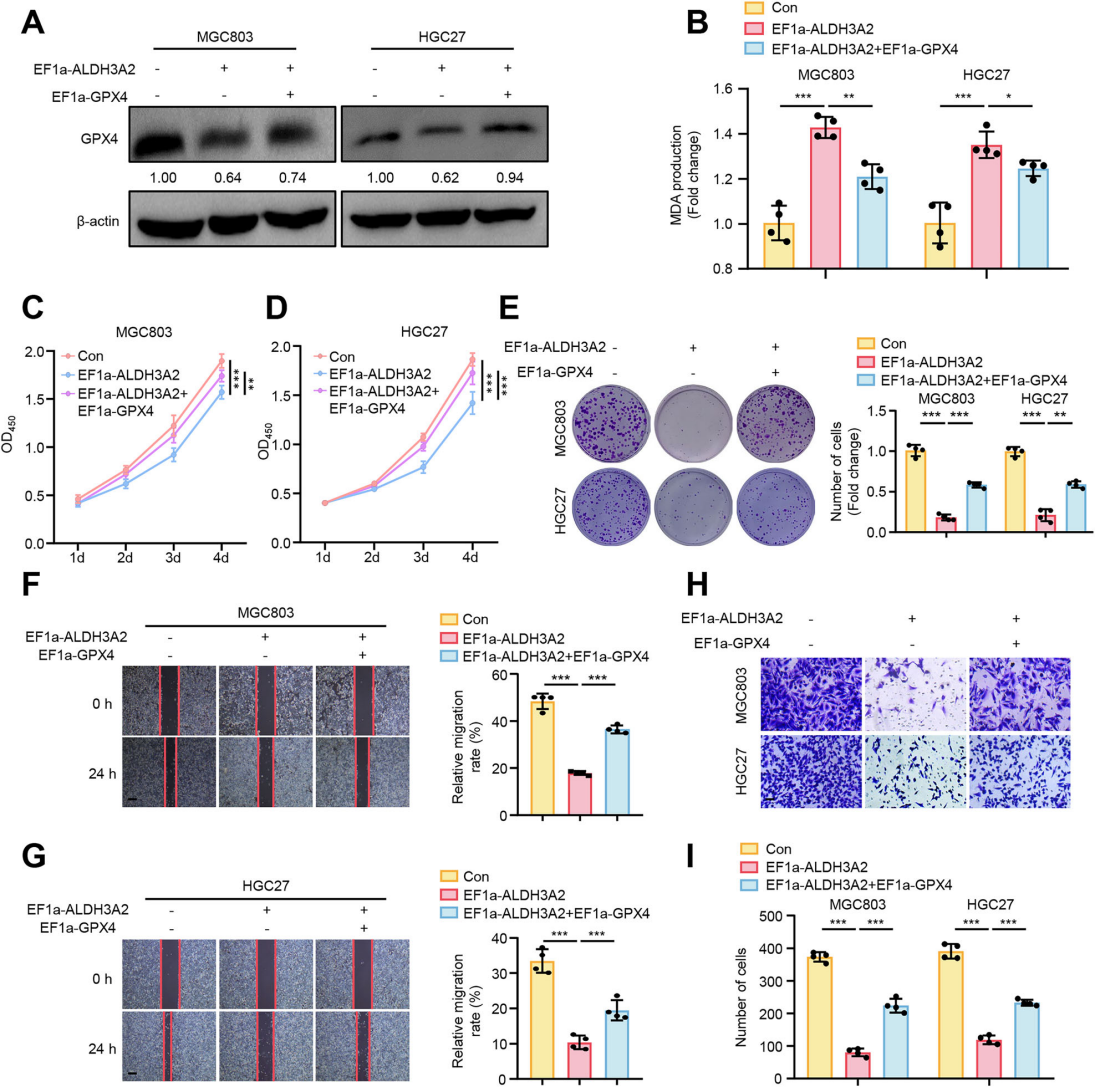
GPX4 overexpression reverses the suppressive effects of ALDH3A2 on malignant progression in GC cells. MGC803 and HGC27 cells overexpressing ALDH3A2 were transduced with PLV-EF1a empty lentivirus (Con), PLV-EF1a-ALDH3A2 alone, or PLV-EF1a-ALDH3A2 together with pLV-EF1a-GPX4. **A** Immunoblotting analysis of GPX4 protein expression with quantitative densitometry (n = 4). **B** MDA levels in GC cells (n = 4). **C**, **D** CCK-8 assay (n = 8) and **E** colony formation assay (n = 4) assessing cell proliferation. **F**, **G** Wound-healing assay and **H**, **I** transwell assay evaluating cell migration and invasion (n = 4). Statistical significance was determined by one-way ANOVA. Data are presented as mean ± SEM. *p < 0.05, **p < 0.01, ***p < 0.001. Scale bar = 0.1 mm.

To determine whether the suppressive effects of ALDH3A2 are mediated through GPX4-dependent ferroptosis, we overexpressed GPX4 to counteract ferroptosis in ALDH3A2-overexpressing MGC803 and HGC27 cell lines. As expected, the enhanced ferroptosis provoked by ALDH3A2 overexpression was partially blocked by GPX4 overexpression (Fig. 3A), as evidenced by decreased MDA levels (Fig. 3B) and increased cell viability (Fig. 3C, D) compared to the ALDH3A2-overexpression group. Notably, GPX4 overexpression significantly rescued ALDH3A2-mediated decreases in cell proliferation (Fig. 3E), migration (Fig. 3F, G), and invasion (Fig. 3H, I) in both MGC803 and HGC27 cells. These data indicate that ALDH3A2 inhibits the aggressive progression of GC cells by promoting ferroptosis in a GPX4-dependent manner.

### ALDH3A2 induces ferroptosis by promoting mitochondrial dysfunction in GC cells

**Fig. 4:**
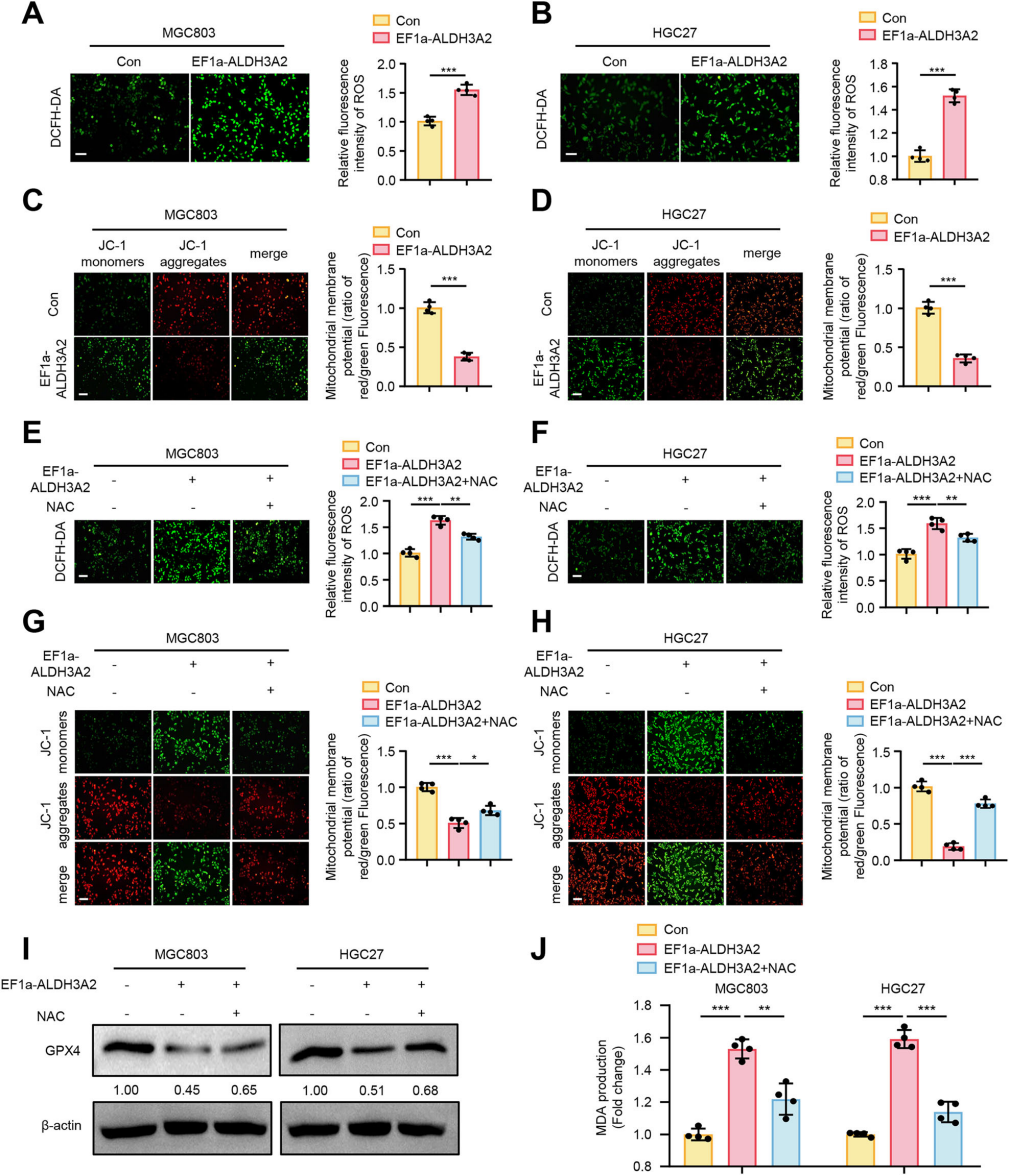
ALDH3A2 promotes ferroptosis by exacerbating mitochondrial dysfunction in GC cells. MGC803 and HGC27 cells were transduced with either pLV-EF1a as a control (Con) or pLV-EF1a-ALDH3A2 for ALDH3A2 overexpression (EF1a-ALDH3A2). GC cells were treated with the mitochondrial function restorer N-Acetylcysteine (20 µM for 24 h) or DMSO as a control. **A**, **B**, **E**, **F** ROS assays were performed in MGC803 and HGC27 cells (n = 4). **C**, **D**, **G**, **H** Mitochondrial membrane potential assays were conducted in MGC803 and HGC27 cells (n = 4). **I** Immunoblotting analysis of GPX4 expression with quantification shown below the blots (n = 4). **J** Measurement of MDA content (n = 4). Statistical differences were determined using one-way ANOVA. Data are presented as mean ± SEM. *p < 0.05, **p < 0.01, ***p < 0.001. Scale bar = 0.1 mm.

Bioinformatic analysis identified ALDH3A2 as a ferroptosis- and mitochondrial dysfunction-related gene, prompting us to hypothesize that ALDH3A2 modulates GC progression through mitochondrial dysfunction. Consistent with this, DCFH-DA staining revealed that ALDH3A2 overexpression significantly increased ROS production in MGC803 (Fig. 4A) and HGC27 (Fig. 4B) cell lines. Furthermore, ALDH3A2 overexpression resulted in a substantial decrease in mitochondrial membrane potential (Fig. 4C, D). These results demonstrate that ALDH3A2 exacerbates mitochondrial dysfunction. To investigate whether ALDH3A2 promotes ferroptosis by inducing mitochondrial dysfunction in GC cells, we utilized the ROS scavenger N-acetylcysteine (NAC) to rescue the ALDH3A2-induced mitochondrial dysfunction in MGC803 and HGC27 cells. As expected, NAC treatment (20 µM, 24 h) significantly reversed ALDH3A2-induced increases in ROS generation (Fig. 4E, F) and decreases in mitochondrial membrane potential (Fig. 4G, H), as well as ferroptosis, evidenced by increased GPX4 expression and reduced MDA levels compared to the ALDH3A2-overexpression group (Fig. 4I, J). These findings indicate that ALDH3A2 promotes ferroptosis by exacerbating mitochondrial dysfunction.

### ALDH3A2-mediated UPR^mt^ suppression contributes to mitochondrial dysfunction and ferroptosis

**Fig. 5:**
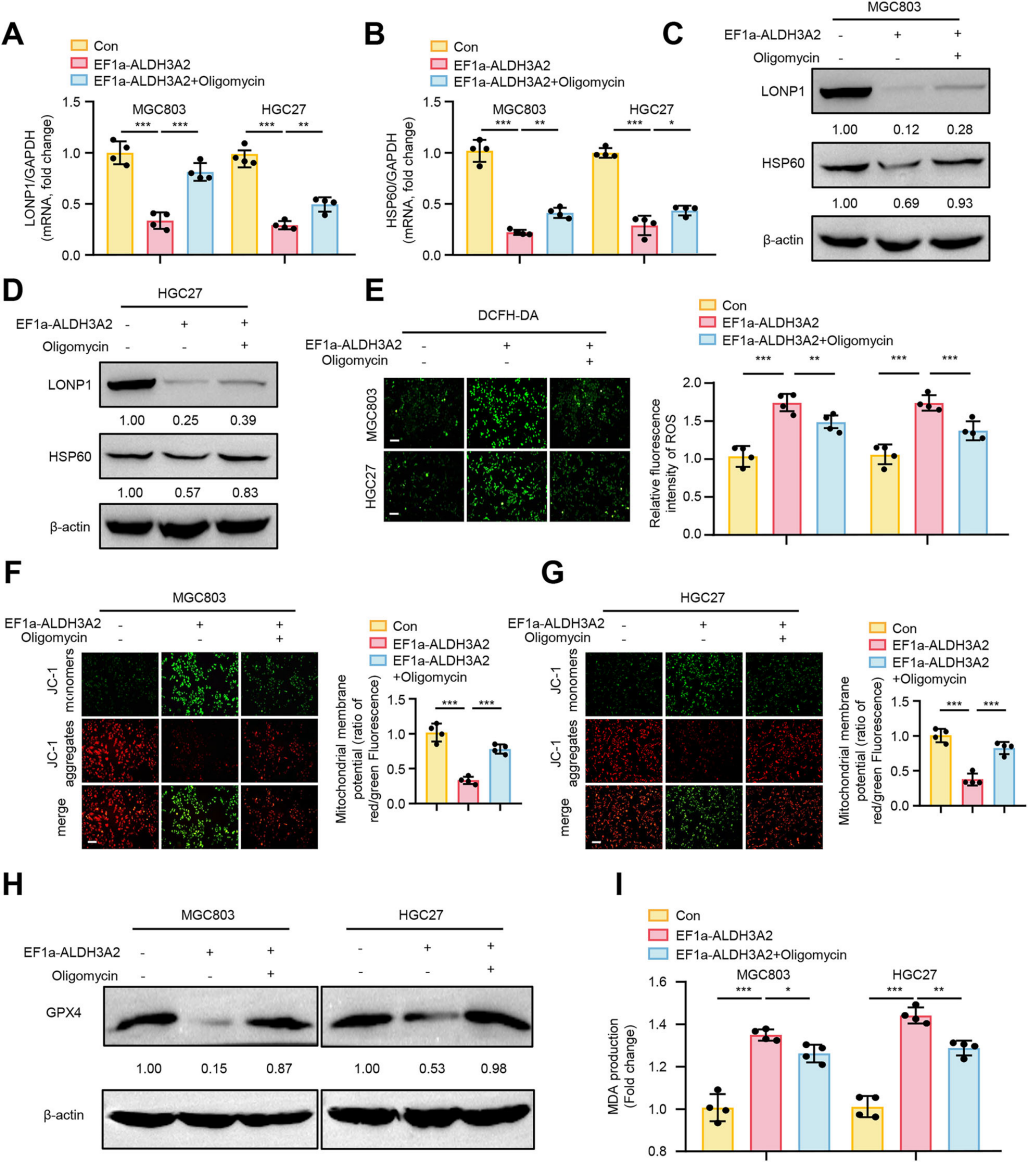
ALDH3A2 drives mitochondrial dysfunction and ferroptosis by impairing UPR^mt^ in GC cells. MGC803 and HGC27 cells were transduced with either pLV-EF1a as a control (Con) or pLV-EF1a-ALDH3A2 for ALDH3A2 overexpression (EF1a-ALDH3A2). GC cells were treated with the UPR^mt^ activator Oligomycin (5 µM for 24 h) or DMSO as a control. **A**, **B** qRT-PCR analysis of LONP1 and HSP60 mRNA expression in MGC803 and HGC27 cells (n = 4). **C**, **D** Immunoblotting analysis of LONP1 and HSP60 protein levels in MGC803 and HGC27 cells, with densitometric quantification normalized to β-actin shown below each blot (n = 4). **E** Intracellular ROS levels (n = 4). **F**, **G** Mitochondrial membrane potential assays in MGC803 and HGC27 cells. **H** Immunoblotting analysis of GPX4 expression with quantification shown below the blots (n = 4). **I** Measurement of MDA content (n = 4). Statistical significance was determined by one-way ANOVA. Data are presented as mean ± SEM. *p < 0.05, **p < 0.01, ***p < 0.001. Scale bar = 0.1 mm.

The UPR^mt^ is essential for maintaining normal mitochondrial function and facilitating mitochondrial recovery [17], which leads us to hypothesize whether ALDH3A2 exacerbates mitochondrial dysfunction by inhibiting UPR^mt^ in GC cells. Remarkably, overexpressing ALDH3A2 significantly reduced both mRNA and protein expression levels of key UPR^mt^ makers LONP1 and HSP60 compared to controls in both MGC803 and HGC27 cells (Fig. 5A–D). Notably, restoration of UPR^mt^ using oligomycin, a known UPR^mt^ activator, was found to attenuate ALDH3A2-induced suppression in UPR^mt^, as evidenced by increased expression of LONP1 and HSP60 in both GC cell lines (Fig. 5A–D). Furthermore, oligomycin-mediated UPR^mt^ restoration was able to reverse ALDH3A2-induced mitochondrial dysfunction and ferroptosis, as demonstrated by reduced ROS accumulation (Fig. 5E), restoration of mitochondrial membrane potential (Fig. 5F, G), upregulation of GPX4 expression (Fig. 5H), and decreased MDA production (Fig. 5I). Functionally, oligomycin treatment effectively rescued ALDH3A2-mediated reduction in cell viability (Fig. S3A, B), proliferation (Fig. S3C), migration (Fig. S3D, E), and invasion (Fig. S3F, G) in GC cells. These results indicate that ALDH3A2 suppresses the UPR^mt^, leading to mitochondrial dysfunction and ferroptosis, which contribute to the suppression of GC progression.

### ALDH3A2 blocking NRF2 nuclear translocation impairs UPR^mt^ to promote mitochondrial dysfunction and ferroptosis

**Fig. 6:**
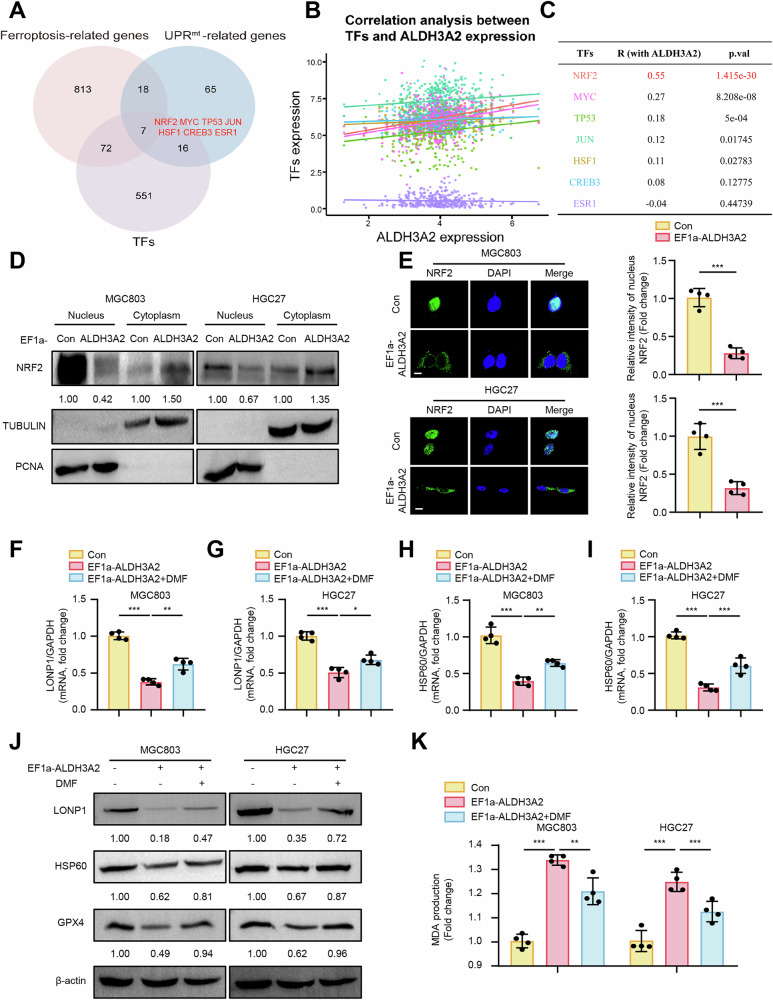
ALDH3A2 impairs UPR^mt^ to exacerbate ferroptosis by blocking NRF2 nuclear translocation in GC cells. MGC803 and HGC27 cells were transduced with either pLV-EF1a as a control (Con) or pLV-EF1a-ALDH3A2 for ALDH3A2 overexpression (EF1a-ALDH3A2). GC cells were treated with the NRF2 activator dimethyl fumarate (DMF, 10 µM, 24 h) or DMSO as a control. **A** Venn diagram illustrating the intersection of transcription factors (TFs), ferroptosis-related genes, and UPR^mt^-related genes. A total of 910 ferroptosis-related genes were obtained from FerrDb, 106 UPR^mt^-related genes from the GeneCards database, and 646 TFs from the hTFtarget database. **B** Pearson correlation analysis and **C** quantification of ALDH3A2 with seven candidate TFs using log2 TPM-normalized RNA-seq data from TCGA-STAD (n = 385). **D** Immunoblot analysis of NRF2 subcellular localization in the nucleus and cytoplasm. TUBULIN and PCNA were used as cytoplasmic and nuclear markers, respectively. Bar graphs show quantification of protein levels (n = 4). **E** Immunofluorescence staining of NRF2 (green) with DAPI nuclear counterstaining (blue). The quantification of NRF2 signals is shown in the right panel (n = 4). Scale bar = 20 µm. **F**–**I** qRT-PCR analysis of LONP1 and HSP60 mRNA expression in MGC803 and HGC27 cells. **J** Immunoblot analysis of LONP1, HSP60, and GPX4 protein expression. Numbers below bands indicate relative protein levels normalized to β-actin (n = 4). **K** Measurement of MDA content (n = 4). Statistical differences were analyzed using one-way ANOVA. Data are presented as mean ± SEM. *p < 0.05, **p < 0.01, ***p < 0.001.

To date, accumulating evidence indicates that transcription factors (TFs) play a well-characterized role in UPR^mt^ regulation in mammalian systems [18–20]. To elucidate the mechanism by which ALDH3A2 suppresses the UPR^mt^ to induce ferroptosis, we conducted an integrative analysis to identify TFs potentially involved in the regulation of ferroptosis and UPR^mt^. Specifically, we intersected three gene sets: 646 TFs from the hTFtarget database, 910 ferroptosis-related genes from the FerrDb database, and 106 UPR^mt^-related genes from the GeneCards database. This analysis identified seven TFs, including NRF2, MYC, TP53, JUN, HSF1, CREB3, and ESR1, as candidates associated with both ferroptosis and UPR^mt^ (Fig. 6A). Subsequently, Pearson correlation analysis was performed between ALDH3A2 and these candidate TFs using RNA-seq expression data from the TCGA-STAD cohort (TPM normalized, n = 385). Among them, NRF2 exhibited the strongest correlation with ALDH3A2 expression (Cor =0.55, p < 0.001) (Fig. 6B, C), suggesting that NRF2 may serve as a key mediator linking ALDH3A2 to ferroptosis and UPR^mt^ regulation. Notably, overexpression of ALDH3A2 did not affect total NRF2 protein levels, as confirmed by immunoblotting (Fig. S4A, B). However, immunoblotting analysis of subcellular fractionation demonstrated that ALDH3A2 significantly reduced NRF2 nuclear translocation in MGC803 and HGC27 cells (Fig. 6D). Immunofluorescence analysis further corroborated these findings, showing diminished NRF2 nuclear translocation in ALDH3A2-overexpressing cells (Fig. 6E). To further evaluate whether NRF2 restoration could reverse ALDH3A2-induced UPR^mt^ impairment and ferroptosis, cells overexpressing ALDH3A2 were treated with dimethyl fumarate (DMF), a known NRF2 activator. Notably, DMF treatment effectively prevented ALDH3A2-induced inhibition of NRF2 nuclear translocation in MGC803 and HGC27 cells (Fig. S4C, D). Functionally, DMF treatment modestly alleviated ALDH3A2-induced UPR^mt^ impairment, as evidenced by elevated mRNA and protein expression of LONP1 and HSP60 (Fig. 6F–J). Moreover, DMF alleviated ferroptosis in ALDH3A2-overexpressing GC cell lines, demonstrated by elevated GPX4 expression (Fig. 6J) and reduced MDA levels (Fig. 6K). Taken together, these findings suggest that one potential mechanism through which ALDH3A2 suppresses the UPR^mt^ could be via the inhibition of NRF2 nuclear translocation, which may in turn contribute to ferroptosis in GC cells.

### ALDH3A2 blocks NRF2 nuclear translocation through downregulation of SLC47A1

**Fig. 7:**
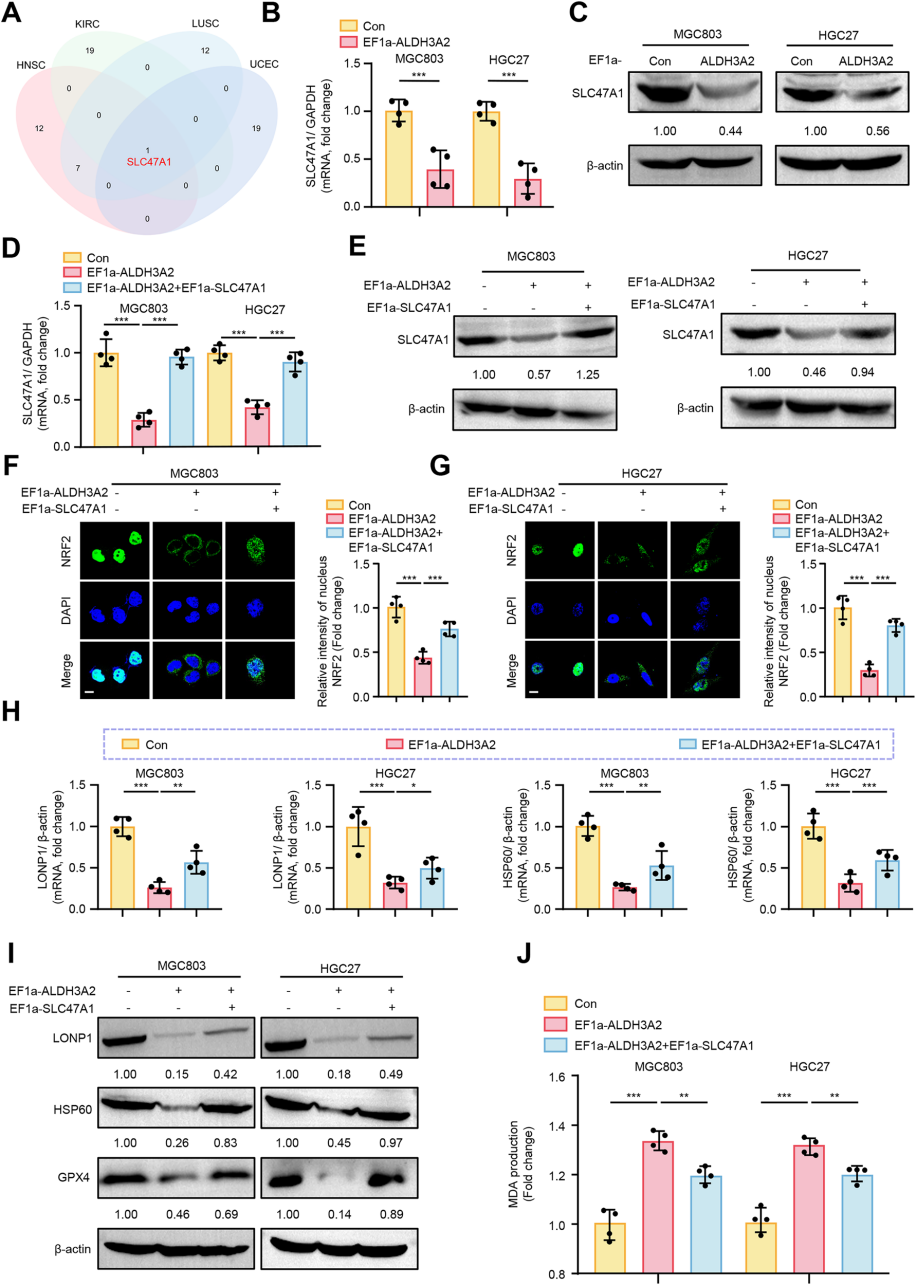
ALDH3A2 blocks NRF2 nuclear translocation via downregulation of SLC47A1 in GC cells. MGC803 and HGC27 cells were transduced with either PLV-EF1a empty lentivirus as a control (Con), PLV-EF1a-ALDH3A2 alone, or PLV-EF1a-ALDH3A2 plus pLV-EF1a-SLC47A1. **A** Venn diagram analysis of ALDH3A2-interacting genes retrieved from the Cancer Cell Metabolism Gene Database (ccmGDB) across head and neck squamous cell carcinoma (HNSC), kidney renal clear cell carcinoma (KIRC), lung squamous cell carcinoma (LUSC), and uterine corpus endometrial carcinoma (UCEC). **B**, **D** qRT-PCR analysis of SLC47A1 mRNA expression in MGC803 and HGC27 cells (n = 4). **C**, **E** Immunoblot analysis of SLC47A1 protein expression in MGC803 and HGC27 cells. Numbers below bands indicate relative protein levels normalized to β-actin (n = 4). **F**, **G** Immunofluorescence staining of NRF2 (green) with DAPI nuclear counterstain (blue) in MGC803 and HGC27 cells. Merged images are shown, and quantification of the NRF2 signal is provided in the right panels (n = 4). Scale bar = 20 µm. **H** qRT-PCR analysis of mitochondrial unfolded protein response markers LONP1 and HSP60 in MGC803 and HGC27 cells (n = 4). **I** Immunoblot analysis of LONP1, HSP60, and GPX4 protein levels, with quantification normalized to β-actin (n = 4). **J** Measurement of MDA content (n = 4). Statistical differences were analyzed using one-way ANOVA. Data are presented as mean ± SEM. *p < 0.05, **p < 0.01, ***p < 0.001.

To further investigate the mechanism by which ALDH3A2 blocks NRF2 nuclear translocation, we queried the Cancer Cell Metabolism Gene Database (ccmGDB) to construct an ALDH3A2-related gene interaction network for identifying the potential downstream target of ALDH3A2 across various cancers. This analysis demonstrated that SLC47A1 was the only common gene interacting with ALDH3A2 in head and neck squamous cell carcinoma (HNSC), kidney renal clear cell carcinoma (KIRC), lung squamous cell carcinoma (LUSC), and uterine corpus endometrial carcinoma (UCEC) (Fig. 7A). These findings prompted us to investigate whether ALDH3A2 suppresses NRF2 nuclear translocation through modulation of SLC47A1 in GC cells. qPCR and immunoblotting analyses demonstrated that ALDH3A2 overexpression significantly reduced SLC47A1 mRNA and protein expression levels in GC cells (Fig. 7B, C). To determine whether ALDH3A2 exerts its biological effects through SLC47A1 downregulation in GC, we overexpressed SLC47A1 in MGC803 and HGC27 cells, which effectively counteracted the ALDH3A2-induced suppression of SLC47A1, as confirmed by qPCR (Fig. 7D) and immunoblotting (Fig. 7E). Immunofluorescence analysis further showed that SLC47A1 overexpression significantly restored NRF2 nuclear translocation, which was reduced by ALDH3A2 overexpression (Fig. 7F, G). Moreover, SLC47A1 overexpression reversed ALDH3A2-induced UPR^mt^ impairment and ferroptosis, as evidenced by increased mRNA and protein levels of LONP1, HSP60, and GPX4, along with decreased MDA levels (Fig. 7H–J). Functionally, SLC47A1 overexpression rescued ALDH3A2-induced inhibition of cell viability (Fig. S5A, B), proliferation (Fig. S5C), migration (Fig. S5D, E), and invasion (Fig. S5F, G) in both GC cell lines. These findings suggest a potential mechanism in which ALDH3A2 downregulates SLC47A1, thereby restricting NRF2 nuclear translocation, impairing UPR^mt^ function, and promoting ferroptosis, which may ultimately contribute to the malignant progression of GC.

### ALDH3A2-induced ferroptosis releasing IL-6 promotes macrophage polarization toward M1 phenotype

**Fig. 8:**
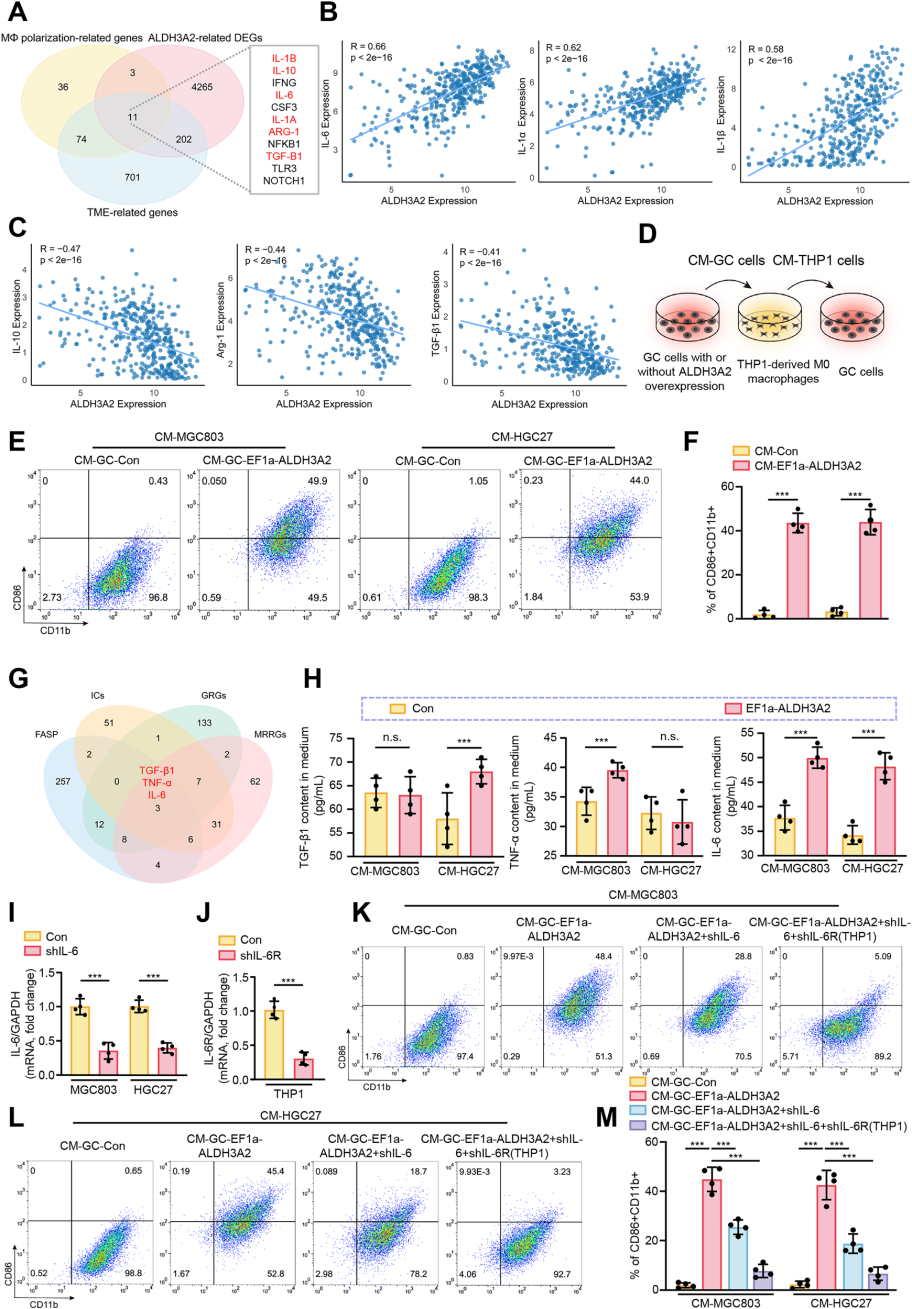
ALDH3A2-induced ferroptosis promotes M1 macrophage polarization via IL-6 secretion. **A** Venn diagram analysis of the overlap among ALDH3A2-related DEGs (derived from the TCGA-STAD cohort, comparing ALDH3A2-high vs. -low groups), macrophage (MΦ) polarization-related genes (GeneCards), and tumor microenvironment (TME)-related genes (TISIDB). Correlation analysis of ALDH3A2 expression with M1 (**B**) and M2 (**C**) macrophage polarization markers. MGC803 and HGC27 cells were transduced with either control lentivirus (PLV-EF1a, Con) or ALDH3A2-overexpressing lentivirus (PLV-EF1a-ALDH3A2). **D** Experimental design: THP-1 cells were incubated with conditioned medium (CM) from MGC803 and HGC27 cells (CM-MGC803, CM-HGC27) for 24 h, followed by co-incubation with freshly cultured MGC803 and HGC27 cells in the presence of CM-treated THP-1 cells (CM-THP-1) for another 24 h. **E**, **F** Flow cytometry analysis of M1 macrophages (CD86⁺CD11b⁺) in THP-1 cells (n = 4). **G** Venn diagram identifying IL-6, TNF-α, and TGF-β1 as overlapping DEGs among ferroptosis-associated secretory phenotype (FASP), inflammatory cytokines (ICs), GC-related genes (GRGs), and macrophage reprogramming-related genes (MRRGs). **H** ELISA measurement of IL-6, TNF-α, and TGF-β1 levels in the culture medium of THP-1 cells exposed to the indicated treatments (n = 4). **I** qRT-PCR analysis of IL-6 mRNA in GC cells transfected with control shRNA (Con) or IL-6 shRNA lentivirus (n = 4). **J** qRT-PCR analysis of IL-6R mRNA in THP-1 cells transfected with control shRNA (Con) or IL-6R shRNA lentivirus (n = 4). **K**–**M** Flow cytometry analysis of M1 macrophage polarization markers in THP-1 cells treated with conditioned medium from GC cells transfected with control, ALDH3A2-overexpressing, or ALDH3A2-overexpressing plus IL-6 shRNA, and/or in the presence of IL-6R knockdown in THP-1 cells (n = 4). Statistical significance was determined by one-way ANOVA. Data are presented as mean ± SEM (n = 4). ***p < 0.001; n.s., not significant.

Tumor-associated macrophages (TAMs) play a crucial role in modulating GC progression via mediating cell proliferation, invasion, and metastasis through TAM polarization [21]. To explore whether ALDH3A2 may modulate TAM polarization, we conducted a novel Venn diagram-based intersection analysis. Specifically, we integrated the DEGs between ALDH3A2-high and -low groups from the TCGA dataset with macrophage polarization-related genes from the GeneCards database and tumor microenvironment (TME)-related genes from the TISIDB database. This analysis identified 11 candidate genes (Fig. 8A). Among these, IL-6 [22], IL-1α [23], and IL-1β [24] are well-established M1 polarization markers, while IL-10 [25, 26], Arg-1 [27], and TGF-β1 [28] are well-recognized M2 polarization markers. Subsequent correlation analyses revealed that ALDH3A2 expression was positively correlated with M1 markers and negatively correlated with M2 markers (Fig. 8B, C), which led us to further hypothesize that ALDH3A2-induced ferroptosis may modulate the tumor microenvironment by promoting TAMs polarization through the release of ferroptosis-associated molecule factors, thereby partially contributing to suppression of GC progression. To validate this hypothesis, we treated THP1-derived M0 macrophages with conditioned medium (CM) from ALDH3A2-overexpressing GC cells (EF1a-ALDH3A2) (Fig. 8D). Flow cytometry analysis revealed that conditioned medium from MGC803 (CM-MGC803) and HGC27 (CM-HGC27) cells overexpressing ALDH3A2 promoted the polarization of THP1-derived M0 macrophages toward the M1 phenotype, as evidenced by a significant increase in the proportion of CD86⁺CD11b⁺ cells (Fig. 8E, F). Consistently, qPCR analysis demonstrated a marked upregulation of M1-associated markers (IL-6, IL-1α, and IL-1β) and a concomitant downregulation of M2-associated markers (IL-10, Arg-1, and TGF-β1) (Fig. S6A). Among these, IL-1β exhibited the most pronounced upregulation. Next, to identify what the ALDH3A2-induced ferroptosis-associated secretory phenotype (FASP) factor promotes macrophage polarization, we conducted a Venn diagram analysis using the GeneCard database to identify protein-coding genes associated with FASP, inflammatory cytokines (ICs), GC-related genes (GRGs), and macrophage reprogramming-related genes (MRRGs). This analysis identified three candidate factors: IL-6, TGF-β1, and TNF-α (Fig. 8G). ELISA assays confirmed that ALDH3A2-induced ferroptosis in both MGC803 and HGC27 cell lines significantly enhanced IL-6 secretion (Fig. 8H). To further validate whether IL-6 released from ALDH3A2-induced ferroptotic GC cells promotes macrophage polarization toward the M1 phenotype, we silenced IL-6 in ALDH3A2-overexpressing GC cells (Fig. 8I) or IL-6 receptor (IL-6R) in THP-1 cells (Fig. 8J). Notably, silencing IL-6 in ALDH3A2-overexpressing GC cells (CM-GC-EF1a-ALDH3A2+shIL-6) or depleting IL-6R in THP-1 cells (CM-GC-EF1a-ALDH3A2+shIL-6+shIL-6R (THP1)) markedly reduced the proportion of M1 macrophages (CD86^+^CD11b^+^) compared with the CM-GC-EF1a-ALDH3A2 group (Fig. 8K–M). Consistently, qPCR analysis revealed that the mRNA expression levels of M1 macrophage markers IL-6, IL-1α, and IL-1β were significantly reduced upon IL-6 or IL-6R knockdown (Fig. S6B), with IL-1β exhibiting the most pronounced reduction. These findings demonstrate that ALDH3A2-induced ferroptosis promotes macrophage polarization toward the M1 phenotype through IL-6 release.

### ALDH3A2-induced ferroptosis promotes macrophage polarization toward the M1 phenotype with elevated IL-1β production to alleviate GC cell progression via PD-L1 downregulation

**Fig. 9:**
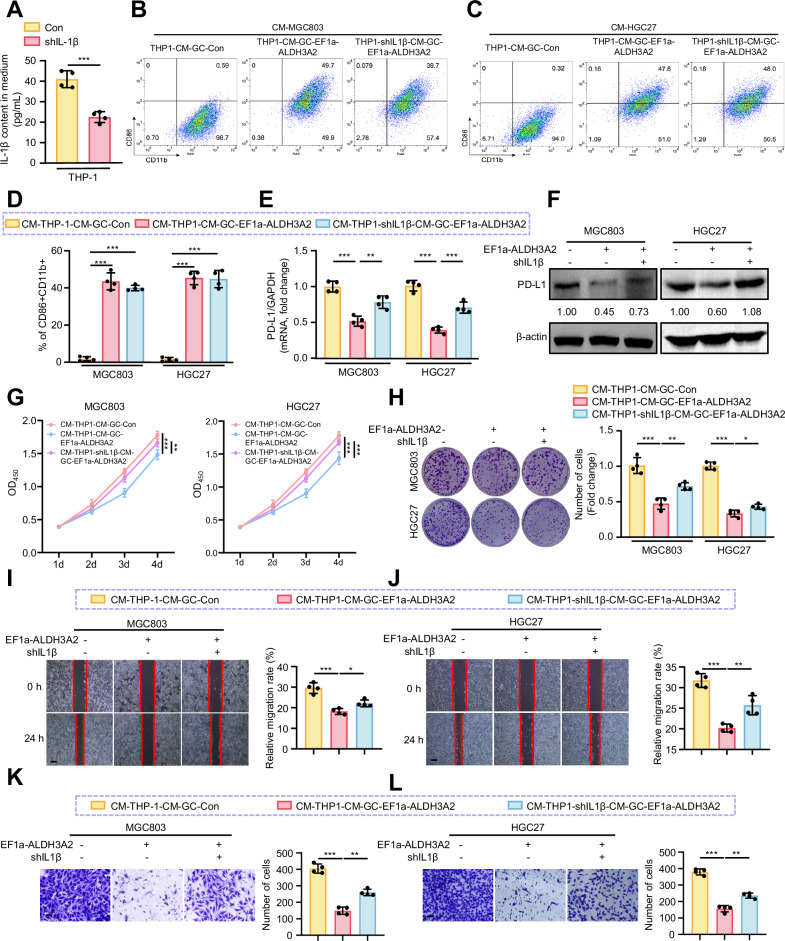
ALDH3A2-induced ferroptosis drives M1 macrophage polarization to suppress GC progression via macrophage-derived IL-1β. THP-1 cells were transfected with scramble shRNA (Con) and IL-1β shRNA lentivirus for 24 h. **A** IL-1β levels in the culture medium of THP-1 cells (n = 4). Freshly cultured GC cells were then incubated with CM-THP-1 for 24 h. **B**–**D** Flow cytometry analysis of M1 macrophage proportions (CD86⁺CD11b⁺) in THP-1 cells (n = 4). **E** qRT-PCR analysis of PD-L1 mRNA expression in MGC803 and HGC27 cells (n = 4). **F** Immunoblot analysis of PD-L1 protein expression in MGC803 and HGC27 cells. Numbers below the bands indicate quantification of PD-L1 levels normalized to β-actin (n = 4). **G** CCK-8 assays (n = 8) and **H** colony formation assays (n = 4) assessing GC cell proliferation. **I**, **J** Wound-healing assays with representative images at 0 h and 24 h after scratching, and quantification of wound closure (n = 4). **K**, **L** Transwell invasion assays (n = 4). Statistical differences were analyzed using one-way ANOVA. Data are presented as mean ± SEM. *p < 0.05, **p < 0.01, ***p < 0.001. Scale bar = 0.1 mm.

Next, we investigated whether the conditioned medium from M1 polarized macrophages modulates the progression of GC through the release of IL-1β. We depleted IL-1β in THP-1 cells to block the IL-1β release from polarized M1 macrophages (Fig. 9A). We first confirmed that THP-1-derived macrophages exposed to conditioned medium from ALDH3A2-overexpressing GC cells (CM-THP1-CM-GC-EF1a-ALDH3A2) exhibited a typical M1 polarization phenotype, as evidenced by an increased proportion of CD86^+^CD11b^+^ cells (Fig. 9B–D). Furthermore, the conditioned medium from these M1-polarized macrophages significantly suppressed the expression of PD-L1, a key factor of immunosuppressive tumor microenvironment, which can be prevented by depleting IL-1β in macrophages (CM-THP1-shIL1β-CM-GC-EF1a-ALDH3A2) (Fig. 9E, F). Functionally, CM-THP1-CM-GC-EF1a-ALDH3A2 significantly reduced cell viability (Fig. 9G), proliferation (Fig. 9H), migration (Fig. 9I, J), and invasion (Fig. 9K, L) in GC cell lines, which can also be reversed by silencing IL-1β in M1 polarized macrophages (CM-THP1-shIL1β-CM-GC-EF1a-ALDH3A2) (Fig. 9G–L). These data suggest that ALDH3A2-induced ferroptosis promotes macrophage polarization toward the M1 phenotype to inhibit GC cell progression via elevation of IL-1β production.

### Targeting ALDH3A2 with genistein attenuates GC progression in vitro and in vivo

**Fig. 10:**
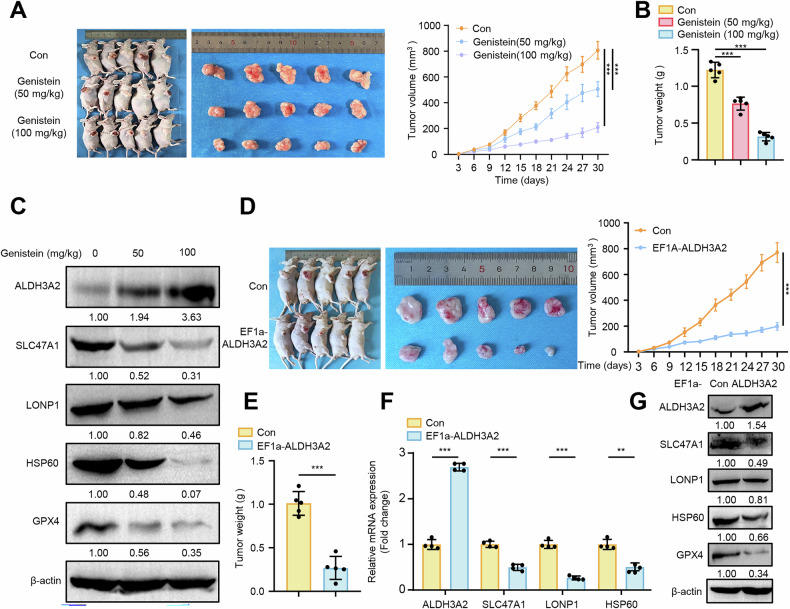
Targeting ALDH3A2 by genistein suppresses tumor growth in vivo. MGC803 cells were subcutaneously injected into nude mice (n = 5), followed by intraperitoneal administration of genistein, and tumor formation was evaluated. In parallel, nude mice were subcutaneously injected with control or ALDH3A2-overexpressing MGC803 cells. Thirty days post-injection, tumors were dissected, photographed, and weighed. Tumor volume was calculated using the formula (length × width²)/2. Representative images of subcutaneous xenograft tumors from control (Con) and genistein-treated groups (50 mg/kg and 100 mg/kg) are shown. Tumor volume was measured every three days after tumor establishment, and tumor weight was recorded at sacrifice. **A** Tumor volumes and **B** tumor weights in each group (n = 5). **C** Immunoblot analysis of ALDH3A2, SLC47A1, LONP1, HSP60, and GPX4 in xenograft tumors, with band intensities quantified relative to β-actin (n = 4). **D** Tumor volumes and **E** tumor weights of control and ALDH3A2-overexpressing groups (n = 5). **F** qRT-PCR analysis of ALDH3A2, SLC47A1, LONP1, and HSP60 mRNA expression in tumor tissues (n = 4). **G** Immunoblot analysis of ALDH3A2, SLC47A1, LONP1, HSP60, and GPX4 protein expression, quantified relative to β-actin (n = 4). Data are presented as mean ± SEM. Statistical significance was determined using one-way ANOVA. **p < 0.01, ***p < 0.001.

The data presented thus far suggest that targeting ALDH3A2 may serve as a promising therapeutic strategy for GC by promoting ferroptosis. Pharmaco-transcriptomic analysis using the DrugBank database identified arsenic trioxide, bezafibrate, bicalutamide, genistein, and rosiglitazone as potential agents capable of upregulating ALDH3A2 expression (Fig. S7A). As shown in Fig. S7B, C, qPCR and immunoblotting analyses revealed that genistein induced the greatest increase in ALDH3A2 mRNA and protein expression in GC cells compared with the other four drug treatments. Furthermore, we observed that treatment with genistein (10 and 20 μM, 48 h) significantly increased ALDH3A2 mRNA levels in GC cells (Fig. S7D). Immunoblotting analysis further confirmed a marked increase in ALDH3A2 expression, accompanied by a reduction in LONP1, HSP60, and GPX4 protein levels in both MGC803 and HGC27 cell lines (Fig. S7E). Additionally, genistein treatment significantly elevated MDA levels, indicating enhanced lipid peroxidation in GC cells (Fig. S7F). DCFH-DA staining shows a significant increase in ROS production after genistein treatment (Fig. S7G). JC-1 staining revealed a substantial reduction in mitochondrial membrane potential following genistein treatment (Fig. S7H, I). Functionally, genistein inhibited GC cell proliferation, as evidenced by CCK-8 (Fig. S7J) and colony formation assays (Fig. S7K) in MGC803 and HGC27 cells. Moreover, wound healing and transwell invasion assays demonstrated that genistein significantly reduced GC cell migration (Fig. S7L, M) and invasion (Fig. S7N). To assess the anti-tumor activity of genistein in vivo, we performed xenograft experiments by implanting MGC803 cells into BALB/c nude mice. Consistently, as shown in Fig. 10A, B, intraperitoneal administration of genistein resulted in a dose-dependent inhibition of MGC803 tumor growth, with the 100 mg/kg genistein group exhibiting significantly greater inhibition compared to both the vehicle and 50 mg/kg genistein groups. Immunoblot analysis of xenograft tumors confirmed that genistein treatment significantly upregulated ALDH3A2 expression while concurrently reducing SLC47A1, LONP1, HSP60, and GPX4 protein expression levels (Fig. 10C). To further verify the role of ALDH3A2 in suppressing GC progression in vivo, MGC803 cells stably transfected with the lentiviral vector EF1a-ALDH3A2 or the empty pLV-EF1a control vector were subcutaneously injected into nude mice. Similarly, tumor volume and weight were significantly reduced in mice injected with ALDH3A2-overexpressing cells compared to control mice (Fig. 10D, E), demonstrating that ALDH3A2 overexpression effectively suppresses GC tumor growth in vivo. qPCR and immunoblotting analyses of tumor tissues isolated from implanted mice revealed that ALDH3A2 overexpression markedly increased the mRNA and protein levels of ALDH3A2 while significantly reducing the mRNA levels of SLC47A1, LONP1, and HSP60 (Fig. 10F) as well as the protein levels of SLC47A1, LONP1, HSP60, and GPX4 (Fig. 10G). These results were consistent with our in vitro findings, further supporting the role of ALDH3A2 in repressing GC growth. Overall, our findings provide compelling evidence that ALDH3A2 overexpression inhibits GC progression by promoting ferroptosis through the ALDH3A2-SLC47A1-UPR^mt^-GPX4 axis, highlighting genistein as a potential therapeutic agent for GC therapy.

## Discussion

Previous bioinformatic investigations suggest that ALDH3A2 is strongly associated with the pathogenesis of GC and may serve as a potential marker for GC diagnosis and prognosis emulation [29, 30]. Consistent with this finding, we provide extensive experimental evidence demonstrating a significant association between aberrant ALDH3A2 expression and the malignant progression of GC in vitro and in vivo. In GC cell lines, depleted ALDH3A2 drives a pro-tumorigenic phenotype, including enhanced cell proliferation, migration, and invasion. Conversely, overexpression of ALDH3A2 in GC cell lines significantly alleviates the aggressive progression of GC cells, suppressing both tumor growth in the xenograft mouse model. Mechanistically, we reveal that ALDH3A2 suppresses pro-carcinogenic progression in GC cells by promoting ferroptosis, thereby inhibiting cell proliferation, migration, and invasion. A similar finding has also been observed in clear cell renal cell carcinoma (ccRCC) [31]. Compared with normal renal tissue, ALDH3A2 expression is significantly downregulated in ccRCC tissue, where it functions to inhibit tumor progression by modulating the PI3K-AKT pathway [31]. Of note, we demonstrate that ALDH3A2 suppresses the proliferation, migration, and invasion of GC cells by promoting ferroptosis in a GPX4-dependent manner, thereby suppressing aggressive tumor progression. Similarly, in ovarian cancer, ALDH3A2 modulates ovarian cancer cell survival via mediating ferroptosis [32]. Moreover, in acute myeloid leukemia (AML) cells, ALDH3A2 has been demonstrated to regulate ferroptosis and contribute to AML drug resistance by controlling cellular oxidative damage [33] and fatty acid synthesis [34]. These findings highlight the emerging role of ALDH3A2 as a key regulator of ferroptosis across multiple cancer types. However, whether ALDH3A2-mediated ferroptosis broadly contributes to the pathogenesis of other malignancies remains to be fully elucidated.

Mitochondria are central to most metabolic pathways, serving as essential organelles involved in energy and biomass production, acting as metabolic sensors, regulating cancer cell death, and initiating signaling pathways associated with cancer cell migration, invasion, metastasis, and treatment resistance [35]. The UPR^mt^, a complex cellular process that is activated when the protein-folding capacity of the mitochondria is overwhelmed, is dysregulated in various cancers and contributes to tumor initiation, growth, metastasis, and therapeutic resistance [36]. In this study, for the first time, we found that ALDH3A2 exacerbates mitochondrial dysfunction by inhibiting UPR^mt^, thereby promoting ferroptosis in GC cells. In contrast, the other isoform of ALDH3A2, aldehyde dehydrogenase 3A1 (ALDH3A1), has been reported to protect mitochondrial function in adult salivary stem/progenitor cells (SSPC) [37]. Genetic deletion or pharmacological inhibition of ALDH3A1 in SSPC leads to a decrease in mitochondrial DNA copy number, structural abnormalities, lower membrane potential, reduced cellular respiration, and elevated ROS levels, leading to decreased survival of murine SSPC [37]. Also, aldehyde dehydrogenase 2 (ALDH2) mitigates mitochondrial dysfunction by promoting PGC-1α-mediated biogenesis in acute kidney injury [38]. These findings indicate the distinct roles of ALDH3A2 in modulating mitochondrial function as compared to other aldehyde dehydrogenase family isozymes.

NRF2, a transcription factor that confers resistance to oxidative stress and ferroptosis, plays a pivotal role in modulating the biological functions of cancer cells by modulating mitochondrial function [39]. NRF2 activation in cancer cells promotes cancer progression and metastasis, while also conferring resistance to chemotherapy and radiotherapy [40–42]. Here, we uncover that elevated ALDH3A2 markedly suppresses NRF2 activation by blocking NRF2 nuclear translocation, thereby inhibiting UPR^mt^ to exacerbate mitochondrial dysfunction with elevated mtROS and reduced mitochondrial membrane potential, which triggers ferroptosis in GC cells. Activated NRF2 has been linked to enhanced antioxidant capacity, chemoresistance, and poor clinical prognosis in GC [43]. In previous investigation, the UPR from endoplasmic reticulum (ER) stress has been found to trigger NRF2 nuclear import in embryonic fibroblasts (MEFs) [44]. Whereas, our results demonstrate that NRF2 nuclear translocation promotes UPR^mt^ activation in GC cells. Similarly, in stressed cardiomyocytes, enhanced nuclear translocation of NRF2 promotes UPR^mt^ activation through its interaction with PGAM5 at the mitochondrial membrane [45]. Furthermore, our results demonstrate that ALDH3A2 inhibits NRF2 activation through downregulation of SLC47A1 expression in GC cells. SLC47A1, a multidrug and toxin extrusion transporter, is thought to facilitate the cellular efflux of oxidative stress-related metabolites [46], which may indirectly support NRF2 activation by maintaining redox balance. Under homeostatic conditions, NRF2 is sequestered in a cytoplasmic complex through its interaction with the actin-binding protein KEAP1 [47]. However, whether SLC47A1 modulates the nuclear translocation of NRF2 in a KEAP1-dependent manner still warrants extensive investigation.

In this study, we reveal that ALDH3A2-induced ferroptosis in GC cells not only suppresses GC tumor progression but also contributes to the reprogramming of the tumor immune microenvironment. Notably, ferroptotic GC cells exhibit elevated secretion of pro-inflammatory cytokines, particularly IL-6, a key mediator of immune responses. The released IL-6 acts in a paracrine manner to promote macrophage polarization toward the M1 phenotype [22]. This polarization shift is critical, as M1 macrophages are associated with anti-tumor immunity, including increased phagocytic activity and the secretion of cytokines that facilitate tumor cell clearance [48]. Mechanistically, we reveal that ALDH3A2-induced ferroptosis promotes macrophage polarization toward the M1 phenotype to alleviate GC cell progression by reducing PD-L1 expression through IL-1β signaling. Similarly, in GC cells, tumor-associated macrophages can promote GC cell proliferation by inducing PD-L1 expression through IL-6 and TNF-ɑ signaling [49]. These findings underscore a dual role for ALDH3A2-induced ferroptosis in GC, directly inducing GC cell ferroptotic death and indirectly inducing anti-tumor immunity via modulation of macrophage function in the tumor microenvironment.

Moreover, we provide robust in vivo evidence demonstrating that targeting ALDH3A2, either through genistein treatment or gene overexpression, effectively inhibits GC cell proliferation, migration, and invasion, while suppressing tumor growth and metastasis by promoting ferroptosis. Genistein, an isoflavone, has been clinically recognized as a promising anticancer agent, showing efficacy in mitigating various cancers, including ovarian cancer [50], liver cancer [51], breast cancer [52], pancreatic cancer [53], head and neck squamous cell carcinoma [54], and oral squamous cell carcinoma [55]. Mechanistically, genistein has been revealed to mitigate cancer progression by promoting apoptosis, regulating the cell cycle, suppressing angiogenesis, and inhibiting metastasis [56]. In GC, substantial evidence also suggests that genistein exhibits significant anti-tumor activity [57–60]. These findings further underscore the clinical potential of genistein as a therapeutic agent for GC treatment through the regulation of ALDH3A2 expression. However, beyond its regulation at the expression level, the enzymatic activity of ALDH3A2 may also play a pivotal role in modulating GC progression. Previous studies have indicated that the dysregulated enzymatic activity of aldehyde dehydrogenases (ALDHs) family member is implicated in cancer pathogenesis [61]. In glioblastoma and squamous cell carcinoma (SCC), abnormal regulation of ALDH3A1 enzymatic activity sensitized tumor cells to ferroptosis through enhanced lipid peroxidation, and a selective enzymatic activity inhibitor of ALDH3A1 (ALDH3A1-EN40) significantly increased ferroptosis sensitivity, thereby suppressing SCC cell proliferation and tumor growth [62]. Similarly, in human glioblastoma, inhibition of ALDH1A3 enzymatic activity by chemical inhibitors protected tumor cells from RSL3-induced ferroptosis by regulating ferritinophagy and intracellular iron release [63]. Functionally, ALDH3A2, like other members of the ALDH family, catalyzes the oxidation of medium- and long-chain aliphatic aldehydes into fatty acids [64]. This enzymatic function orchestrates certain key cellular processes in GC cells, including ATP production [65], fatty acid oxidation [65], oxidative stress and inflammation [66], all of which contribute to GC progression. Thus, altered enzymatic activity of ALDH3A2 may also influence GC progression through detoxification of the toxic aldehydes and regulation of metabolic and oxidative stress pathways. Nevertheless, the specific contribution of ALDH3A2 enzymatic activity to GC progression requires further elucidation. In particular, whether its enzymatic activity and expression levels exert distinct or overlapping roles in modulating tumor growth and ferroptosis warrants further investigation.

## Conclusion

In this study, we identified that ALDH3A2 as a key ferroptosis- and mitochondrial dysfunction-related gene negatively contributes to GC progression. Mechanistically, ALDH3A2 blocks NRF2 nuclear translocation via downregulating SLC47A1, thereby impairing UPR^mt^ activation, which in turn leads to mitochondrial dysfunction and GPX4-dependent ferroptosis. Furthermore, ALDH3A2-induced ferroptosis promotes an anti-tumor immune microenvironment via M1 macrophage polarization and IL-1β-mediated PD-L1 downregulation. Together, these synergistic effects suppress GC cell proliferation, migration, and invasion, ultimately restraining tumor growth.

## Supplementary information

Table 1

Supplementary Figures

Original Western Blot Images

aj-checklist

## Data Availability

The datasets supporting the conclusions of this article are included within the article and its additional files.
