# Supplementary material for: ALDH3A2 negatively orchestrates gastric cancer progression through a synergistic induction of ferroptosis and ferroptosis-driven macrophage reprogramming
Source: Cell Death Dis. 2025 Dec 24;17(1):97. doi: 10.1038/s41419-025-08364-8 (PMC12830774; doi:10.1038/s41419-025-08364-8)
Supplement: Supplementary file 1 — Table 1 [file 41419_2025_8364_MOESM1_ESM.docx]

**Supplemental Table 1. The clinical information of GC cohorts.**

| Features |  | TCGA STADREAD |
| --- | --- | --- |
| Sample Size |  | N = 415 |
| Status | Live | 256 |
|  | Dead | 159 |
| Age | ≥65 | 238 |
|  | <65 | 177 |
| Gender | Male | 147 |
|  | Female | 268 |
| Tumor Stage | Stage I | 59 |
|  | Stage II | 122 |
|  | Stage III | 170 |
|  | Stage IV | 40 |
|  | Unknown | 24 |
| T Stage | T1 | 22 |
|  | T2 | 91 |
|  | T3 | 181 |
|  | T4 | 113 |
|  | Tis | 8 |
| N Stage | N0 | 125 |
|  | N1 | 110 |
|  | N2 | 79 |
|  | N3 | 82 |
|  | NX | 19 |
| M Stage | M0 | 367 |
|  | M1 | 27 |
|  | MX | 21 |
