# Supplementary material for: ALDH3A2 negatively orchestrates gastric cancer progression through a synergistic induction of ferroptosis and ferroptosis-driven macrophage reprogramming
Source: Cell Death Dis. 2025 Dec 24;17(1):97. doi: 10.1038/s41419-025-08364-8 (PMC12830774; doi:10.1038/s41419-025-08364-8)
Supplement: Supplementary file 2 — Supplementary Figures [file 41419_2025_8364_MOESM2_ESM.pdf]

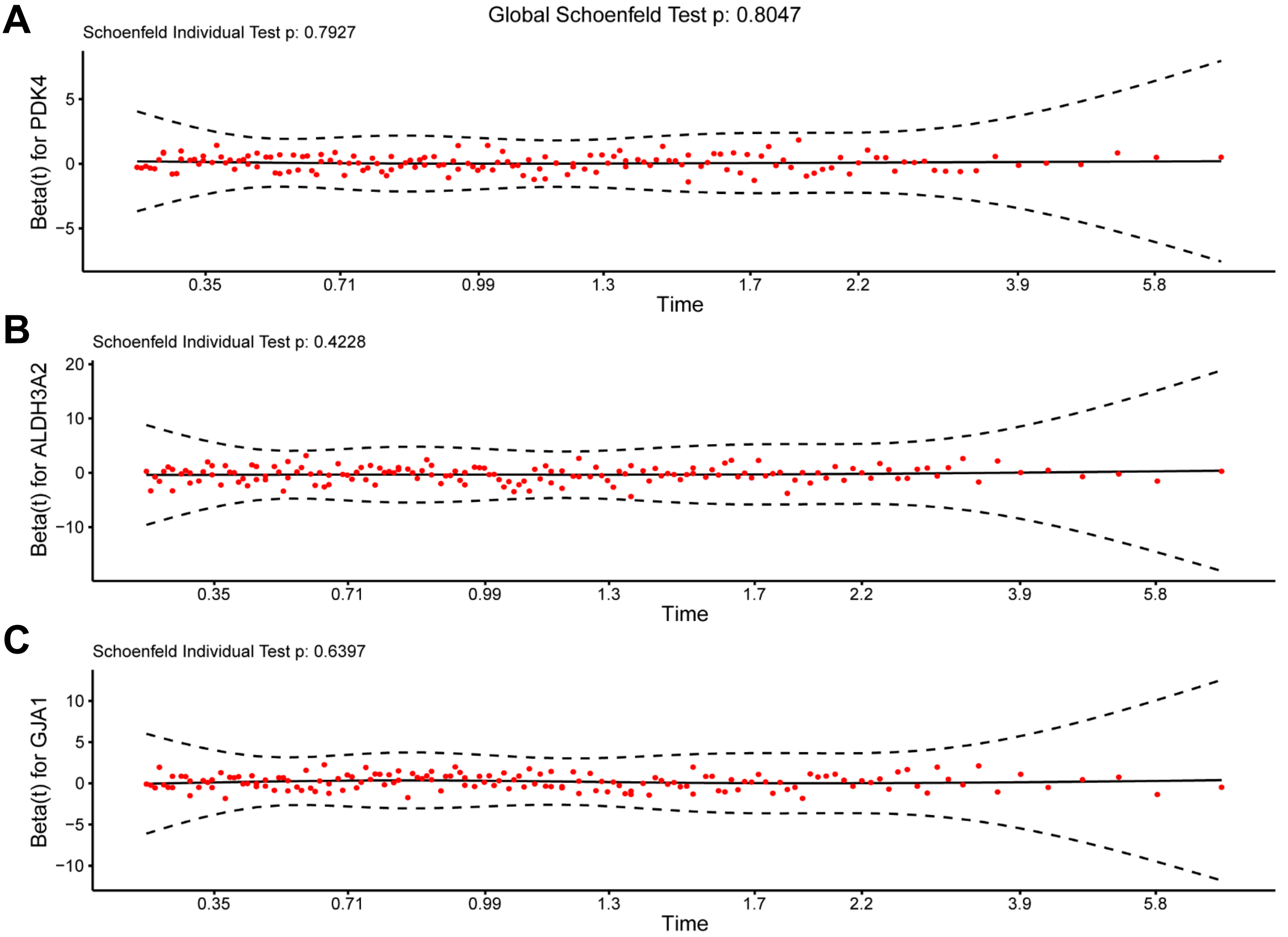

Fig. S1 Schoenfeld residual analysis for Cox model covariates. Schoenfeld residuals plots for PDK4 (A), ALDH3A2 (B), and GJA1 (C) display residuals (red dots) against time with fitted lines (solid) and 95% confidence intervals (dashed). The global Schoenfeld test ($p = 0.8047$) and individual p-values (PDK4: 0.7927; ALDH3A2: 0.4228; GJA1: 0.6397) indicate no violation of the proportional hazards assumption.

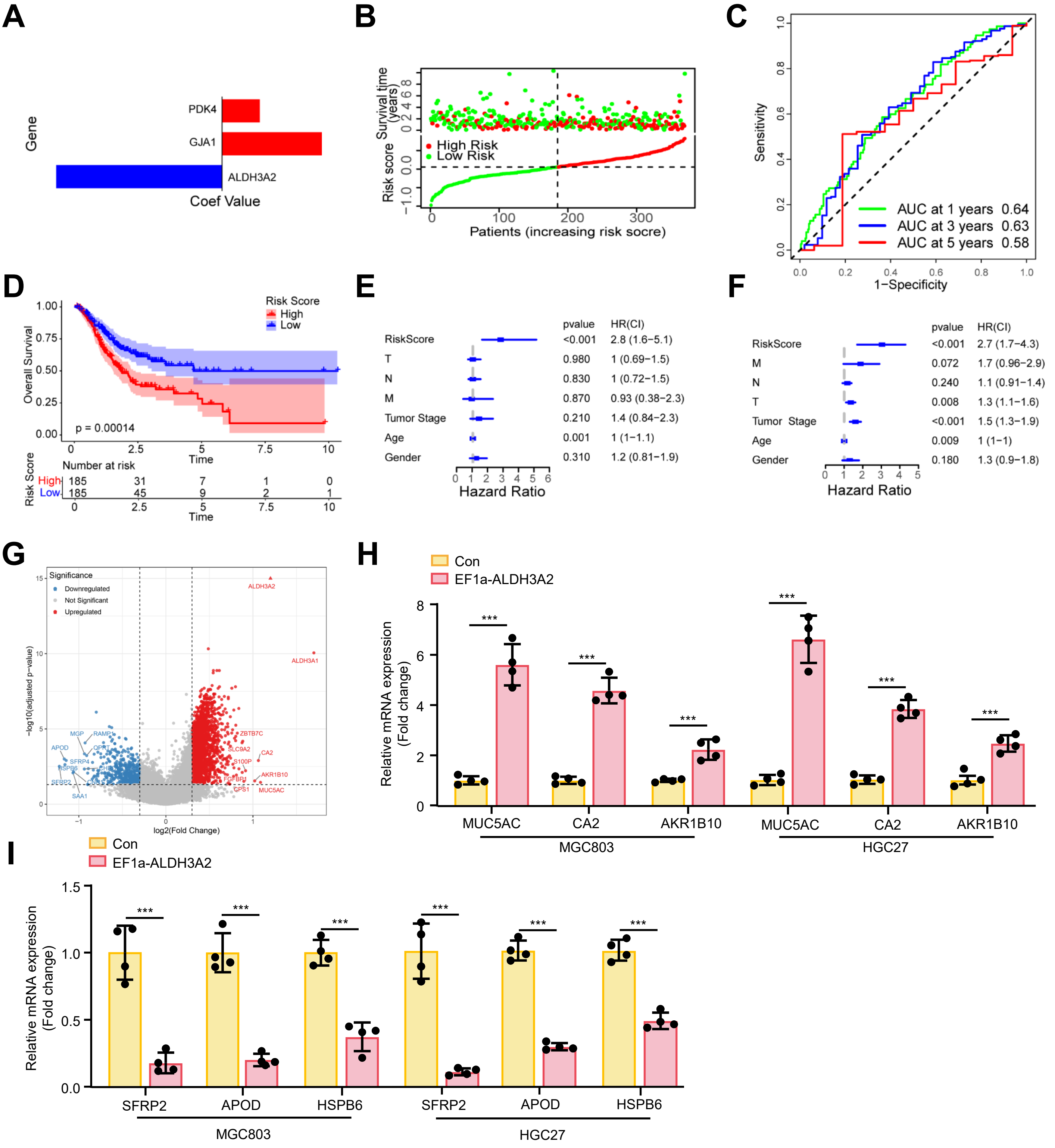

Fig. S2 Prognostic value and validation of the risk model based on PDK4, ALDH3A2, and GJA1 in GC. (A) Coefficients of the PDK4, ALDH3A2, and GJA1 in the prognostic model. (B) Risk score distribution and classification of patients into high- and low-risk groups. (C) ROC curves for 1-, 3-, and 5-year survival predictions. (D) Kaplan–Meier survival curves of overall survival between high- and low-risk groups. (E–F) Univariate and multivariate Cox regression analyses of overall survival. (G) Volcano plot of differentially expressed genes between ALDH3A2-high and -low groups in TCGA-STAD (|log2FoldChange| > 1 and adjusted p < 0.05). Red and blue dots represent upregulated and downregulated genes, with the top 10 labeled. (H–I) qRT-PCR analysis of MUC5AC, CA2, AKR1B10, SFRP2, APOD, and HSPB6 mRNA expression in MGC803 and HGC27 cells (n = 4). Statistical significance was determined by an unpaired Student's t-test. Data are presented as mean ± SEM. ***p < 0.001. Scale bar, 0.1 mm.

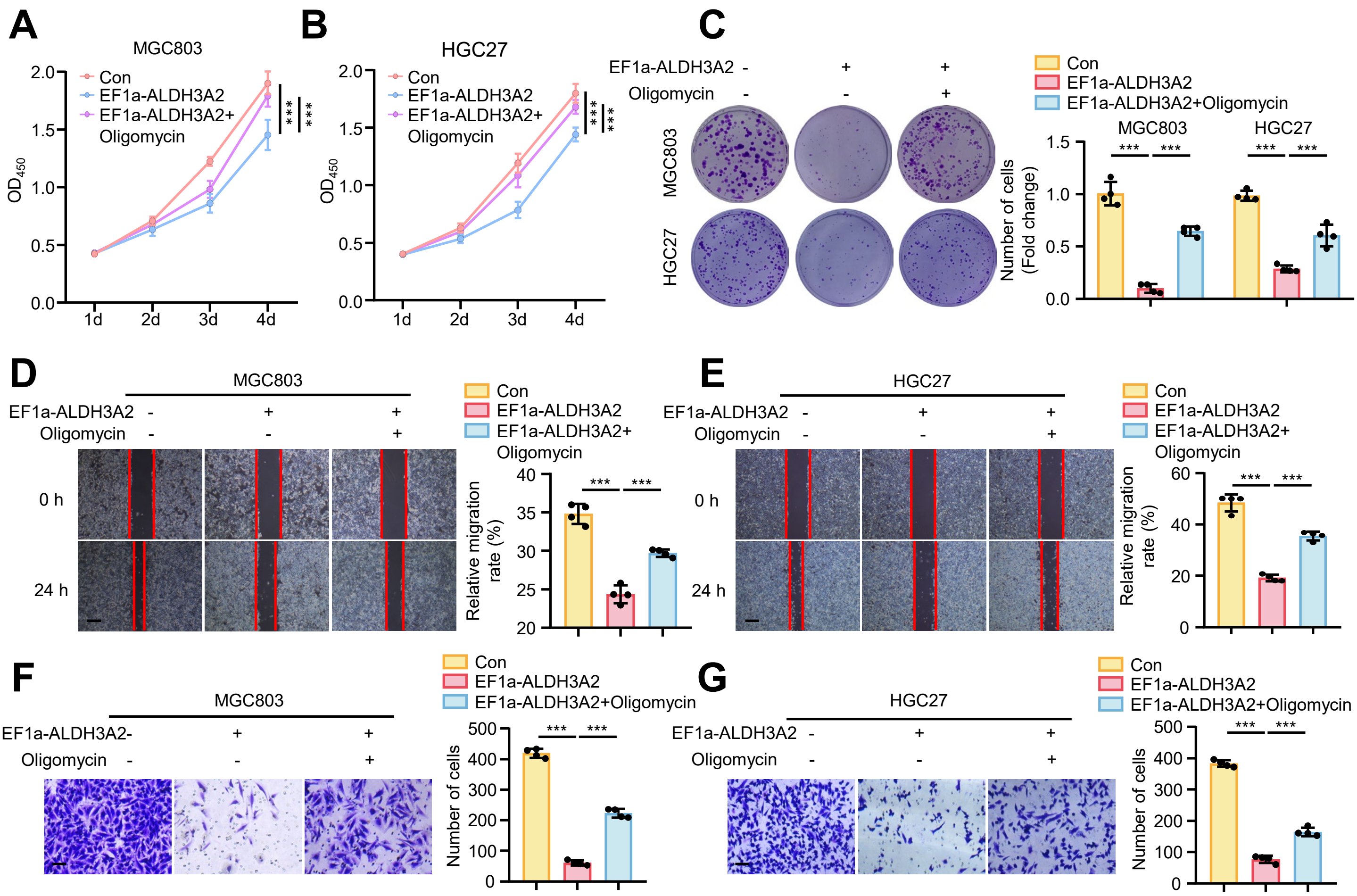

Fig. S3 Oligomycin counteracts the inhibitory effects of ALDH3A2 on GC cells progression. MGC803 and HGC27 cells overexpressing ALDH3A2 were transduced with PLV-EF1a empty lentivirus (Con) or pLV-EF1a-ALDH3A2 for ALDH3A2 overexpression (EF1a-ALDH3A2). GC cells were treated with the UPR^{mt} activator Oligomycin (5 μM, 24 h) or DMSO as a control. (A-B) CCK-8 assays (n = 8) and (C) colony formation assays (n = 4) assessing cell proliferation. (D-E) Wound-healing assays and (F-G) transwell assays evaluating cell migratory and invasive (n = 4). Statistical significance was determined by one-way ANOVA. Data are presented as mean ± SEM. ***p < 0.001. Scale bar = 0.1 mm.

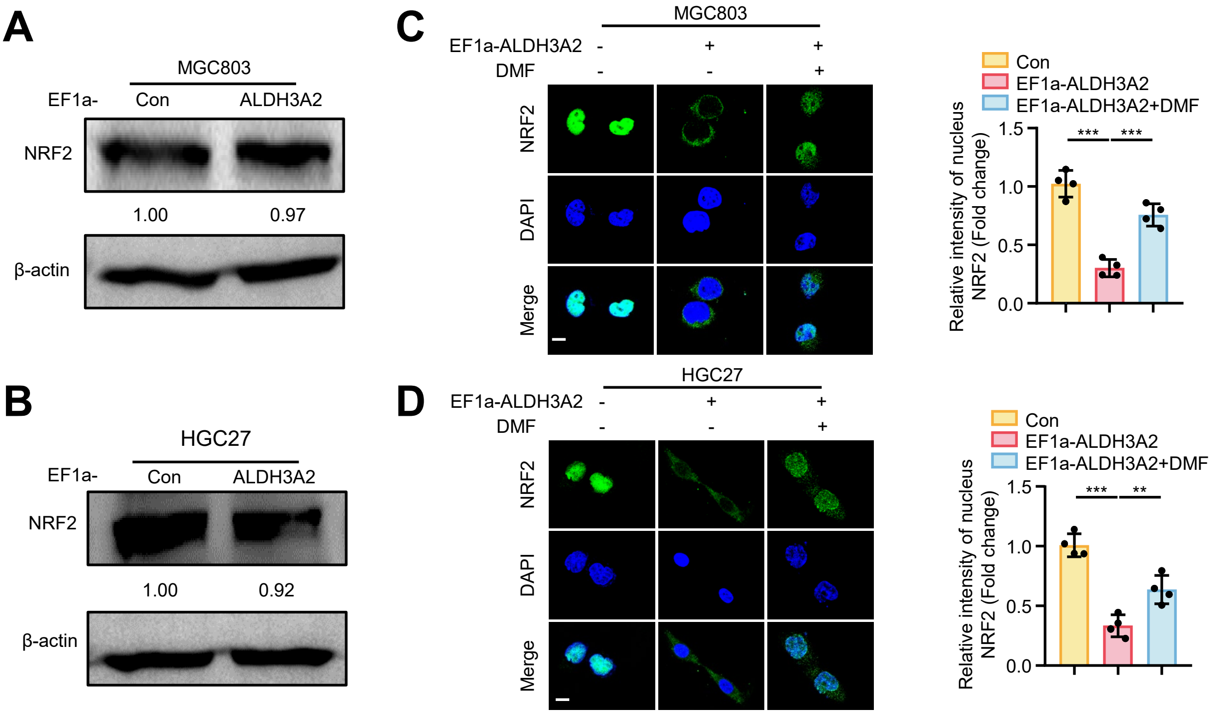

Fig. S4 ALDH3A2 overexpression suppresses NRF2 nuclear translocation without affecting its total expression in GC cells. MGC803 and HGC27 cells overexpressing ALDH3A2 were transduced with PLV-EF1a empty lentivirus (Con) or pLV-EF1a-ALDH3A2 for ALDH3A2 overexpression (EF1a-ALDH3A2). GC cells were treated with the NRF2 activator DMF (10 μM for 24 h) or DMSO as a control. (A-B) Immunoblotting analysis of NRF2 protein expression with quantitative densitometry (n = 4). (C, D) Immunofluorescence staining of NRF2 (green) with DAPI nuclear counterstaining (blue). The quantification of NRF2 signals is shown in the right panel (n = 4). Scale bar = 20 μm.** $p < 0.01$, *** $p < 0.001$, n.s.: not significant.

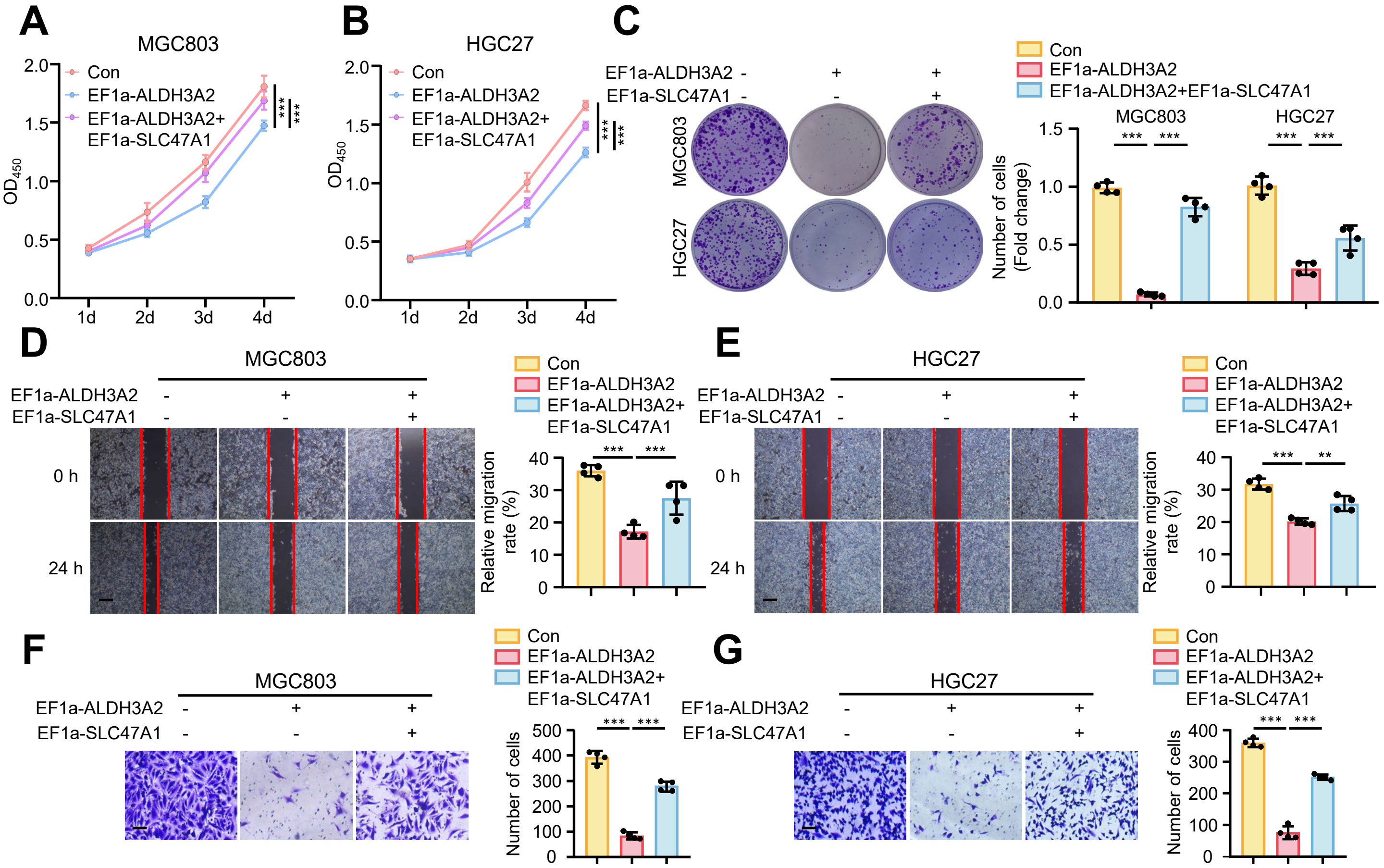

Fig. S5 SLC47A1 overexpression abrogates the suppressive effects of ALDH3A2 on malignant progression in GC cells. MGC803 and HGC27 cells overexpressing ALDH3A2 were transduced with PLV-EF1a empty lentivirus (Con), PLV-EF1a-ALDH3A2 alone, or PLV-EF1a-ALDH3A2 together with pLV-EF1a-SLC47A1. (A-B) CCK-8 assay (n = 8) and (C) colony formation assays (n = 4) were performed to assess cell proliferation. (D-E) Wound-healing assays and (F-G) Transwell assays were conducted to evaluate the migratory and invasive capabilities of GC cells (n = 4). Statistical differences were determined using one-way ANOVA. Data are presented as mean ± SEM. **p < 0.01, ***p < 0.001. Scale bar, 0.1 mm.

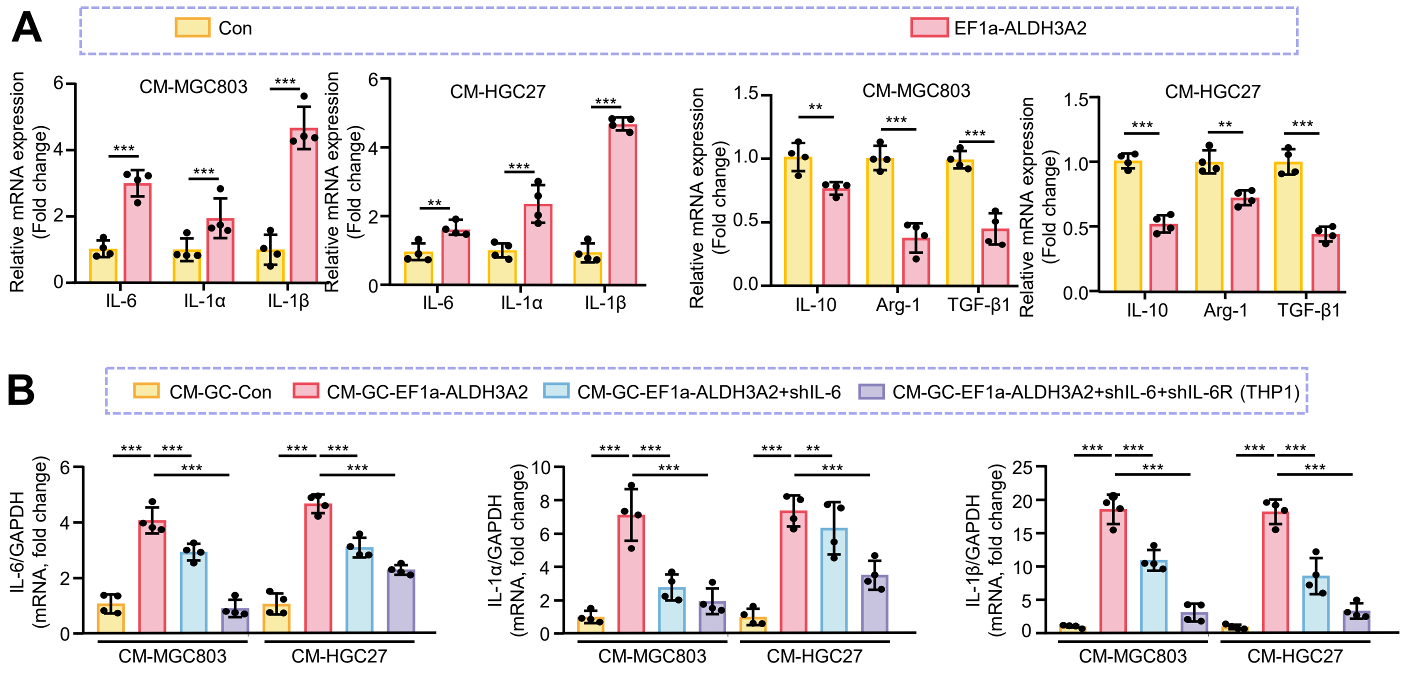

Fig. S6 ALDH3A2-induced ferroptosis enhances M1 macrophage marker expression through IL-6 secretion. MGC803 and HGC27 cells with ALDH3A2 overexpression were transduced with either pLV-EF1a empty lentivirus as a control (Con), pLV-EF1a-ALDH3A2 alone, or pLV-EF1a-ALDH3A2 plus IL-6 shRNA lentivirus. (A) qRT-PCR analysis of IL-6, IL-1α, IL-1β, IL-10, Arg-1, and TGF-β1 mRNA expression in THP-1 cells (n = 4). (B) qRT-PCR analysis of IL-6, IL-1α and IL-1β mRNA expression in THP-1 cells (n = 4). Statistical significance was analyzed using one-way ANOVA. Data are presented as mean ± SEM. ** $p < 0.01$, *** $p < 0.001$.

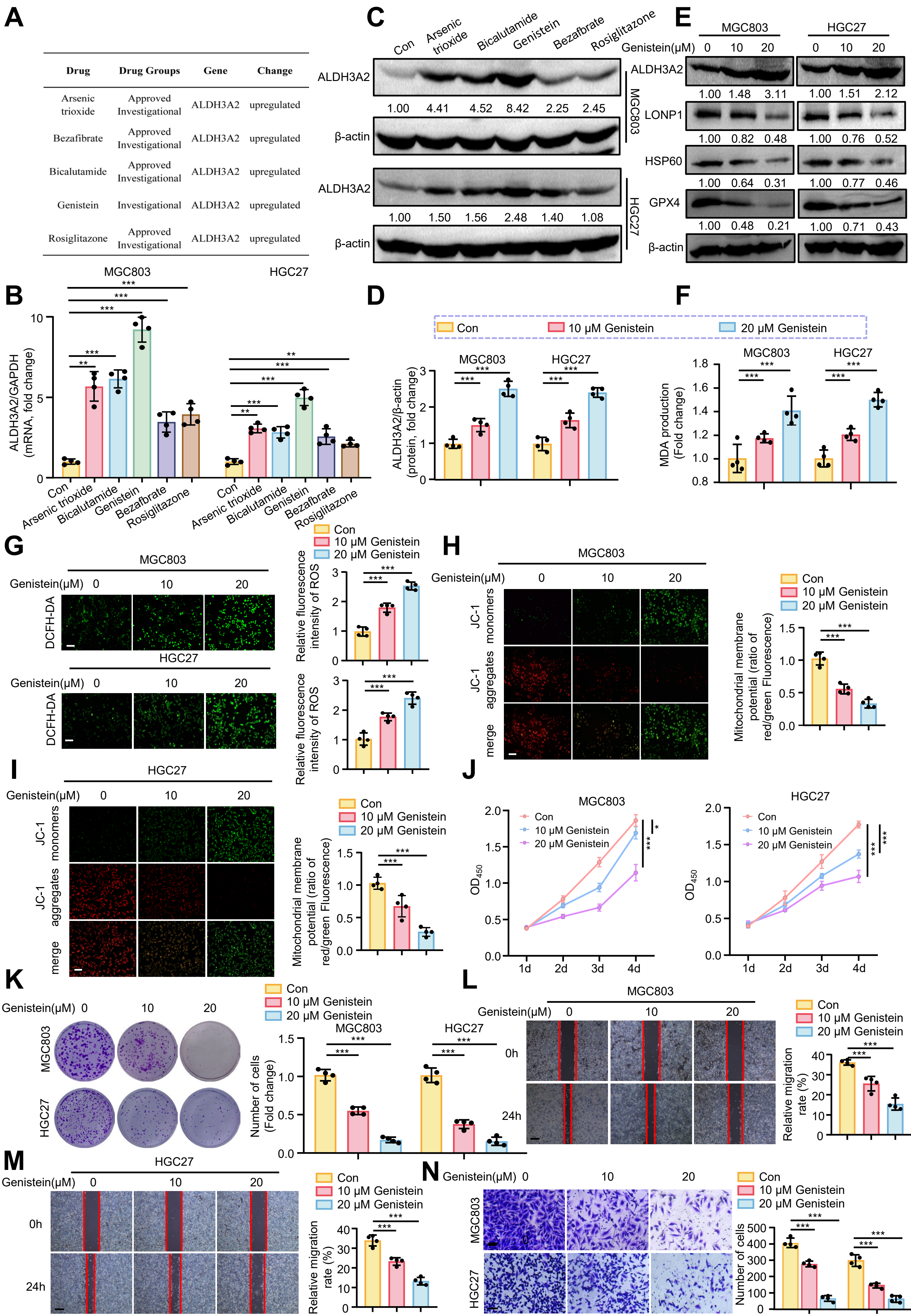

A
Drug | Drug Groups | Gene | Change
Arsenic trioxide | Approved Investigational | ALDH3A2 | upregulated
Bezafibrate | Approved Investigational | ALDH3A2 | upregulated
Bicalutamide | Approved Investigational | ALDH3A2 | upregulated
Genistein | Investigational | ALDH3A2 | upregulated
Rosiglitazone | Approved Investigational | ALDH3A2 | upregulated
B
MGC803
HGC27
ALDH3A2/GAPDH (mRNA, fold change)
Con
Arsenic trioxide
Bicalutamide
Genistein
Bezafibrate
Rosiglitazone
C
Con
Arsenic trioxide
Bicalutamide
Genistein
Bezafibrate
Rosiglitazone
ALDH3A2
β-actin
1.00 4.41 4.52 8.42 2.25 2.45
1.00 1.50 1.56 2.48 1.40 1.08
MGC803
HGC27
D
ALDH3A2/β-actin (protein, fold change)
E
MGC803
HGC27
Genistein(μM) 0 10 20 0 10 20
ALDH3A2
1.00 1.48 3.11 1.00 1.51 2.12
LONP1
1.00 0.82 0.48 1.00 0.76 0.52
HSP60
1.00 0.64 0.31 1.00 0.77 0.46
GPX4
1.00 0.48 0.21 1.00 0.71 0.43
β-actin
F
Con
10 μM Genistein
20 μM Genistein
MDA production (Fold change)
G
Genistein(μM) 0 10 20
DCFH-DA
Relative fluorescence intensity of ROS
H
JC-1 monomers
JC-1 aggregates
merge
Mitochondrial membrane potential (ratio of red/green Fluorescence)
I
J
OD450
1d 2d 3d 4d
K
Number of cells (Fold change)
L
0h
24h
Relative migration rate (%)
M
N
Number of cells

Fig. S7 Genistein upregulates ALDH3A2 expression to drive ferroptosis and inhibit GC cells progression. MGC803 and HGC27 cells were treated with genistein (10 μM , 20 μm and 40 μM) for 48 hours. (A) The expression activators of ALDH3A2 were screened through drug transcriptomic analysis using the DrugBank database. (B,D) qRT-PCR analysis of ALDH3A2 mRNA expression (n = 4). (C) Immunoblotting analysis of ALDH3A2 protein levels in GC cells (n = 4). (E) Immunoblotting analysis of ALDH3A2, LONP1, HSP60 and GPX4 expression (n = 4). (F) Measurement of MDA content (n = 4). (G) ROS assays were performed in MGC803 and HGC27 cells (n = 4). (H-I) Mitochondrial membrane potential assays were conducted in MGC803 and HGC27 cells (n = 4). (J) CCK-8 assays (n = 8) and (K) colony formation assays (n = 4) were performed to assess cell proliferation. (L-M) Wound-healing assays and (N) transwell assays evaluating cell migration and invasion (n = 4). Statistical significance was determined by one-way ANOVA. Data are presented as mean ± SEM. * $p < 0.05$, ** $p < 0.01$, *** $p < 0.001$. Scale bar = 0.1 mm.
