# Supplementary material for: ALDH3A2 negatively orchestrates gastric cancer progression through a synergistic induction of ferroptosis and ferroptosis-driven macrophage reprogramming
Source: Cell Death Dis. 2025 Dec 24;17(1):97. doi: 10.1038/s41419-025-08364-8 (PMC12830774; doi:10.1038/s41419-025-08364-8)
Supplement: Supplementary file 3 — Original Western Blot Images [file 41419_2025_8364_MOESM3_ESM.pdf]

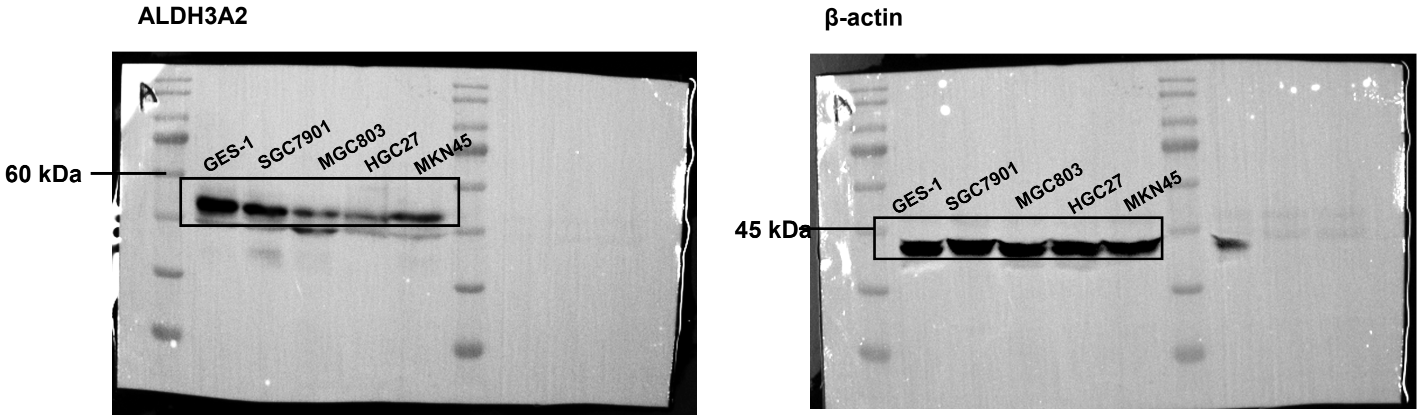
ALDH3A2
β-actin
60 kDa
45 kDa
GES-1
SGC7901
MGC803
HGC27
MKN45

Fig. 1K

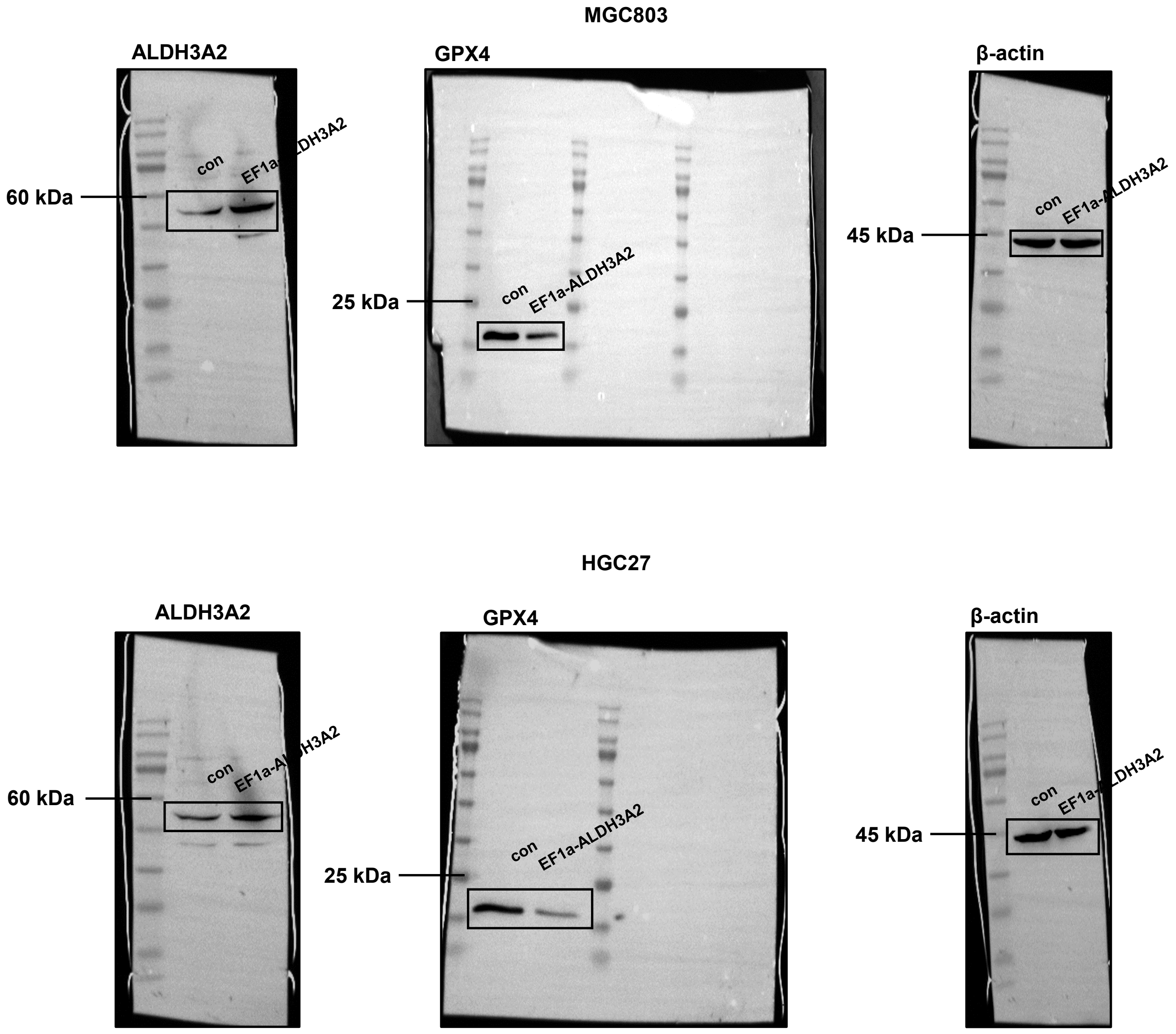
MGC803
ALDH3A2
GPX4
β-actin
60 kDa
25 kDa
45 kDa
con
EF1a-ALDH3A2
HGC27
ALDH3A2
GPX4
β-actin
60 kDa
25 kDa
45 kDa
con
EF1a-ALDH3A2

Fig. 2B

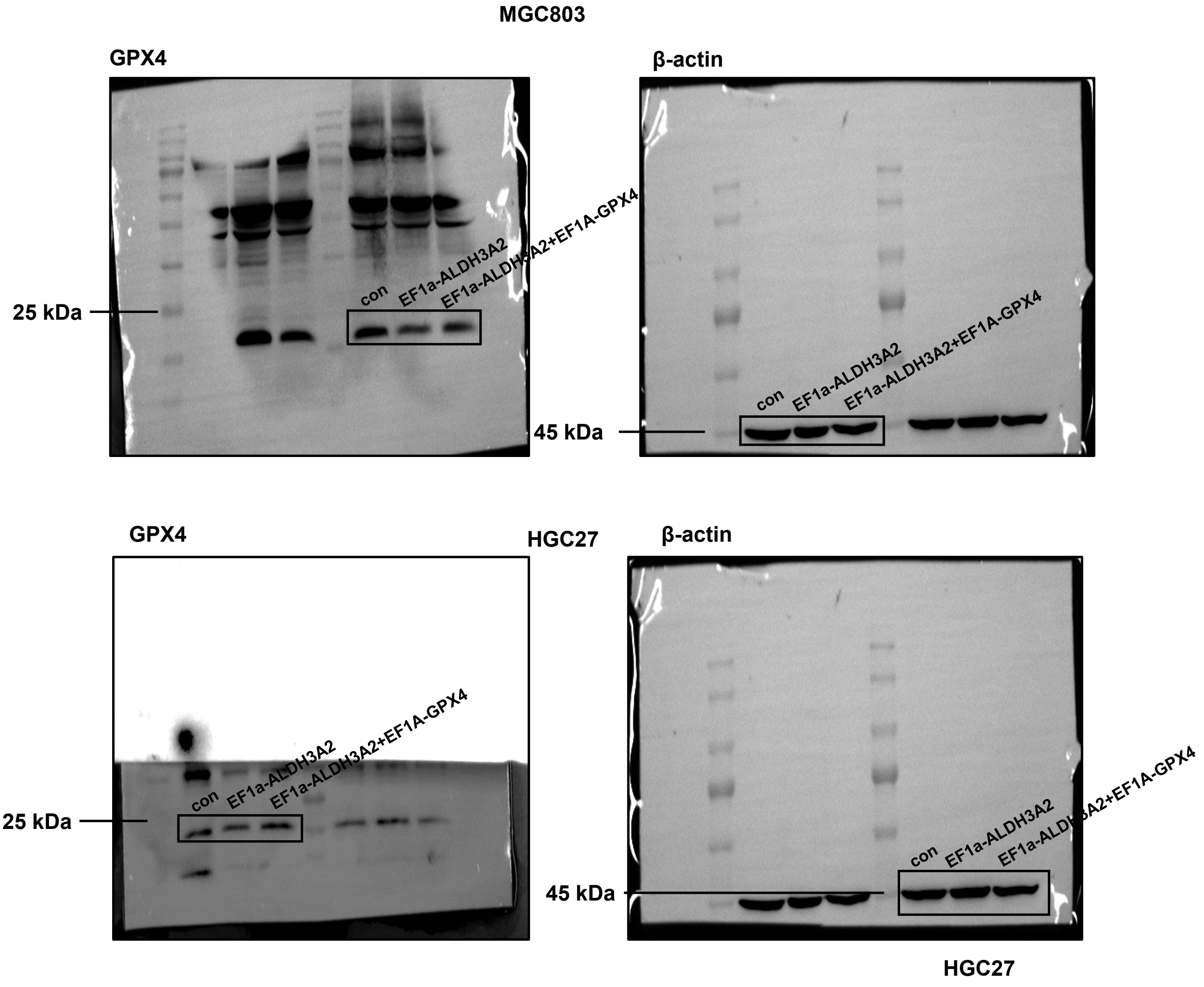
MGC803
GPX4
β-actin
25 kDa
con
EF1a-ALDH3A2
EF1a-ALDH3A2+EF1A-GPX4
45 kDa
GPX4
HGC27
β-actin
25 kDa
con
EF1a-ALDH3A2
EF1a-ALDH3A2+EF1A-GPX4
con
EF1a-ALDH3A2
EF1a-ALDH3A2+EF1A-GPX4
45 kDa
HGC27

Fig. 3A

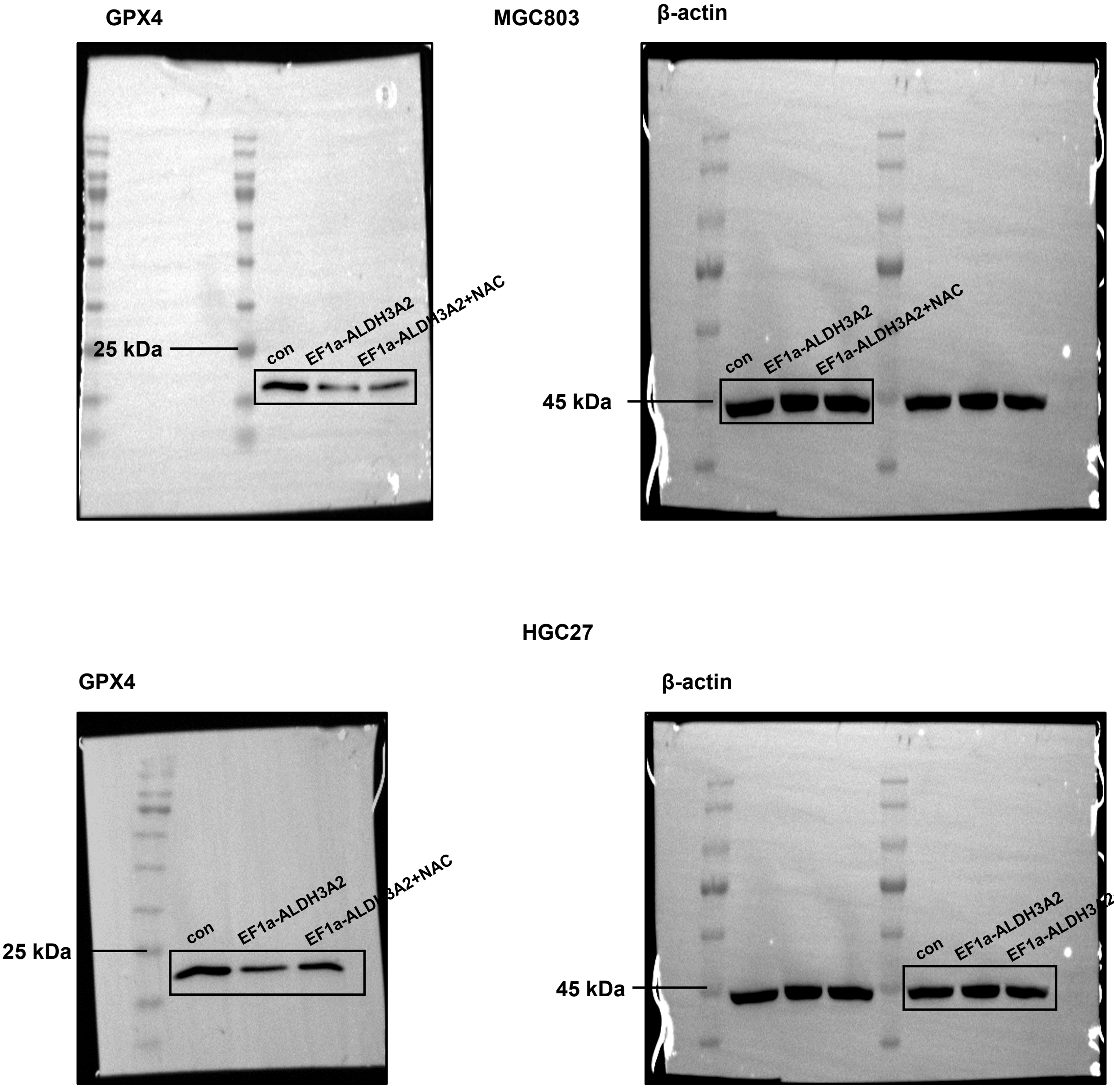
GPX4
MGC803
β-actin
25 kDa
con
EF1a-ALDH3A2
EF1a-ALDH3A2+NAC
45 kDa
con
EF1a-ALDH3A2
EF1a-ALDH3A2+NAC
HGC27
GPX4
β-actin
25 kDa
con
EF1a-ALDH3A2
EF1a-ALDH3A2+NAC
45 kDa
con
EF1a-ALDH3A2
EF1a-ALDH3A2+NAC

Fig. 4I

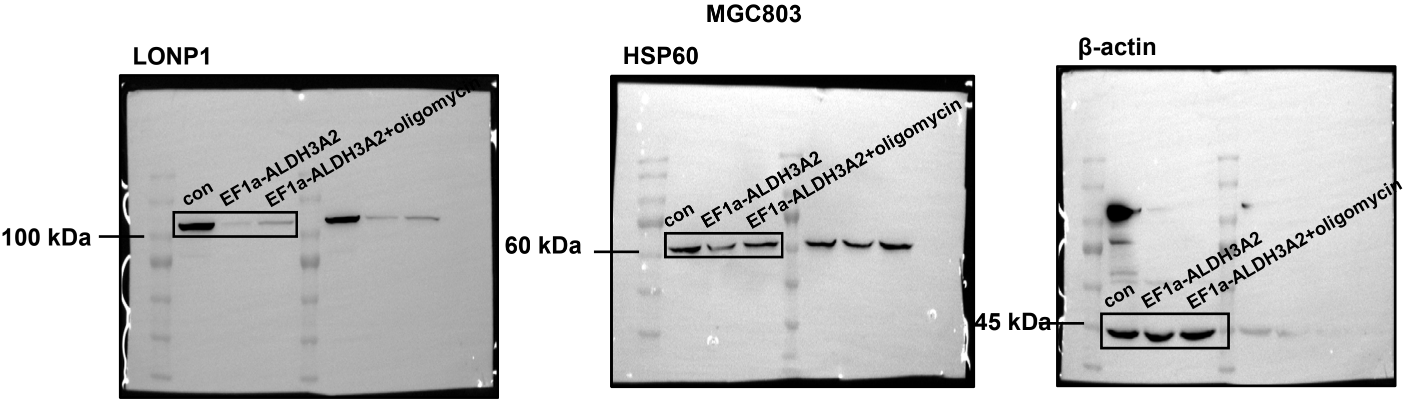

Fig. 5C

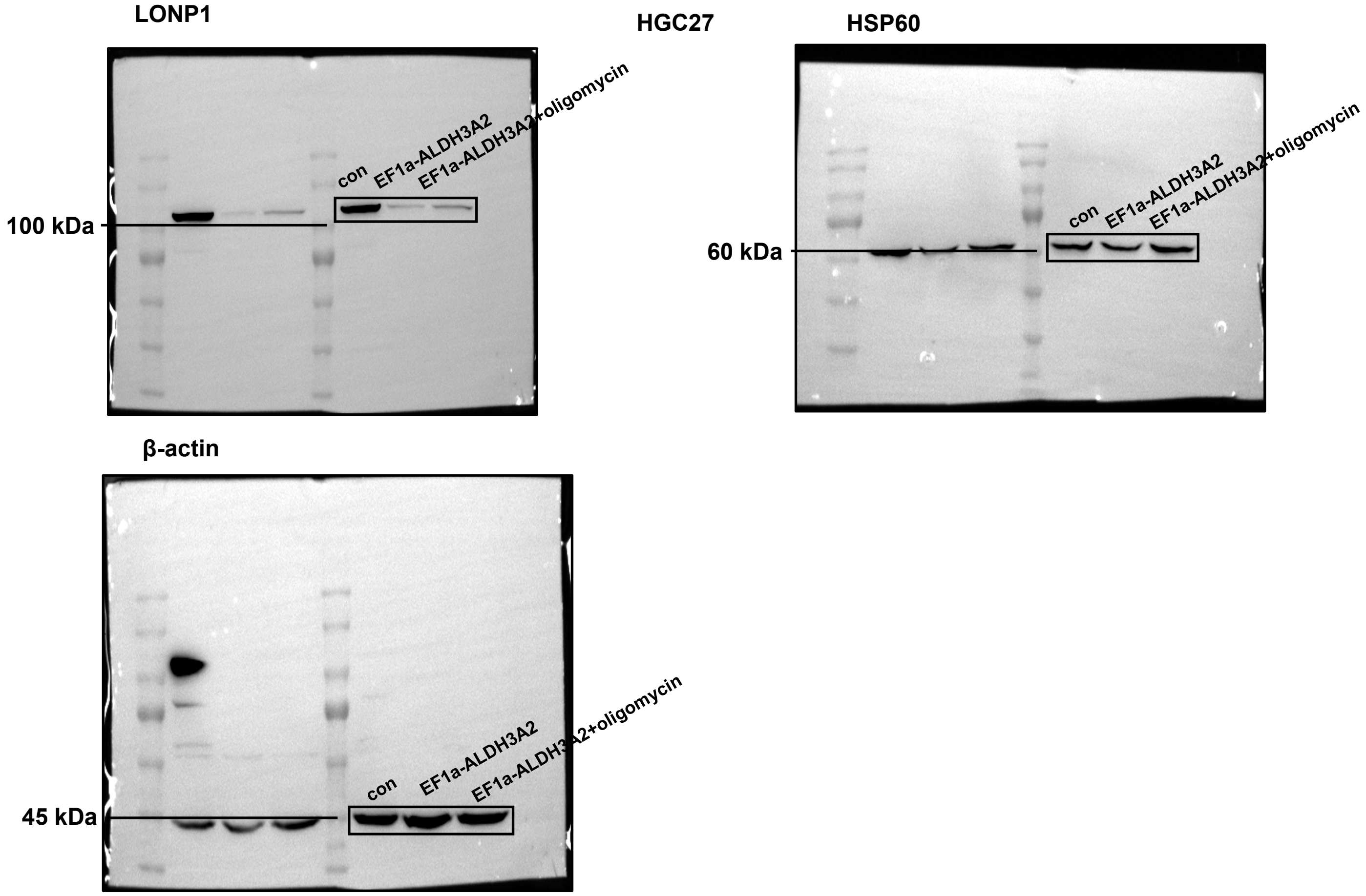

Fig. 5D

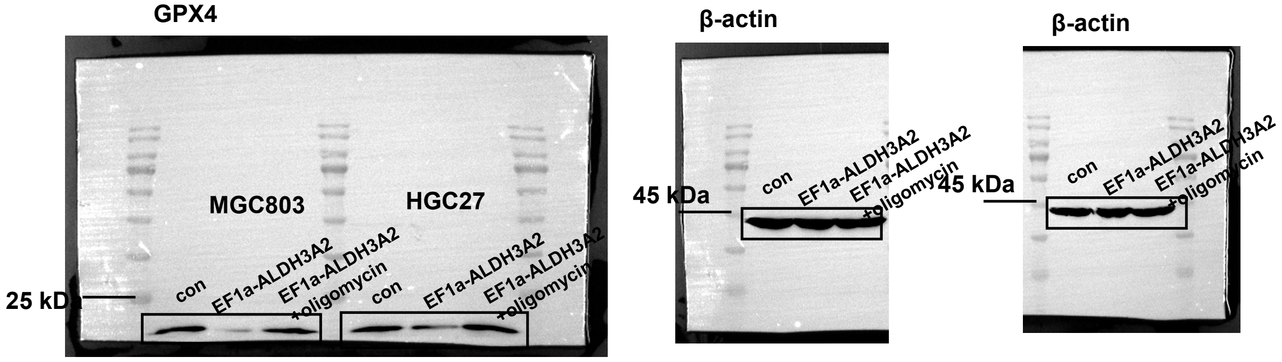

Fig. 5H

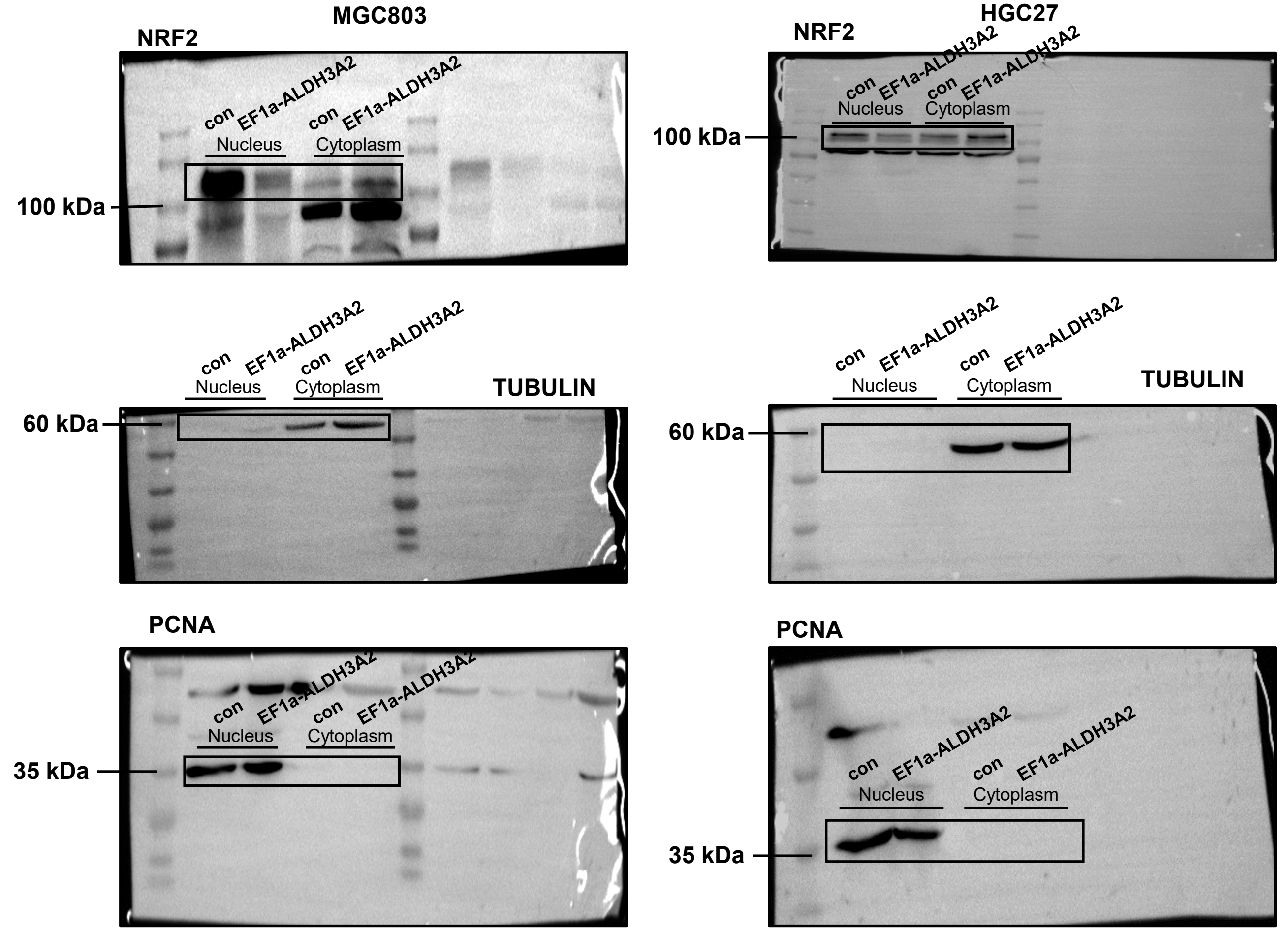
MGC803
HGC27
NRF2
con
EF1a-ALDH3A2
Nucleus
Cytoplasm
100 kDa
TUBULIN
60 kDa
PCNA
35 kDa

Fig. 6D

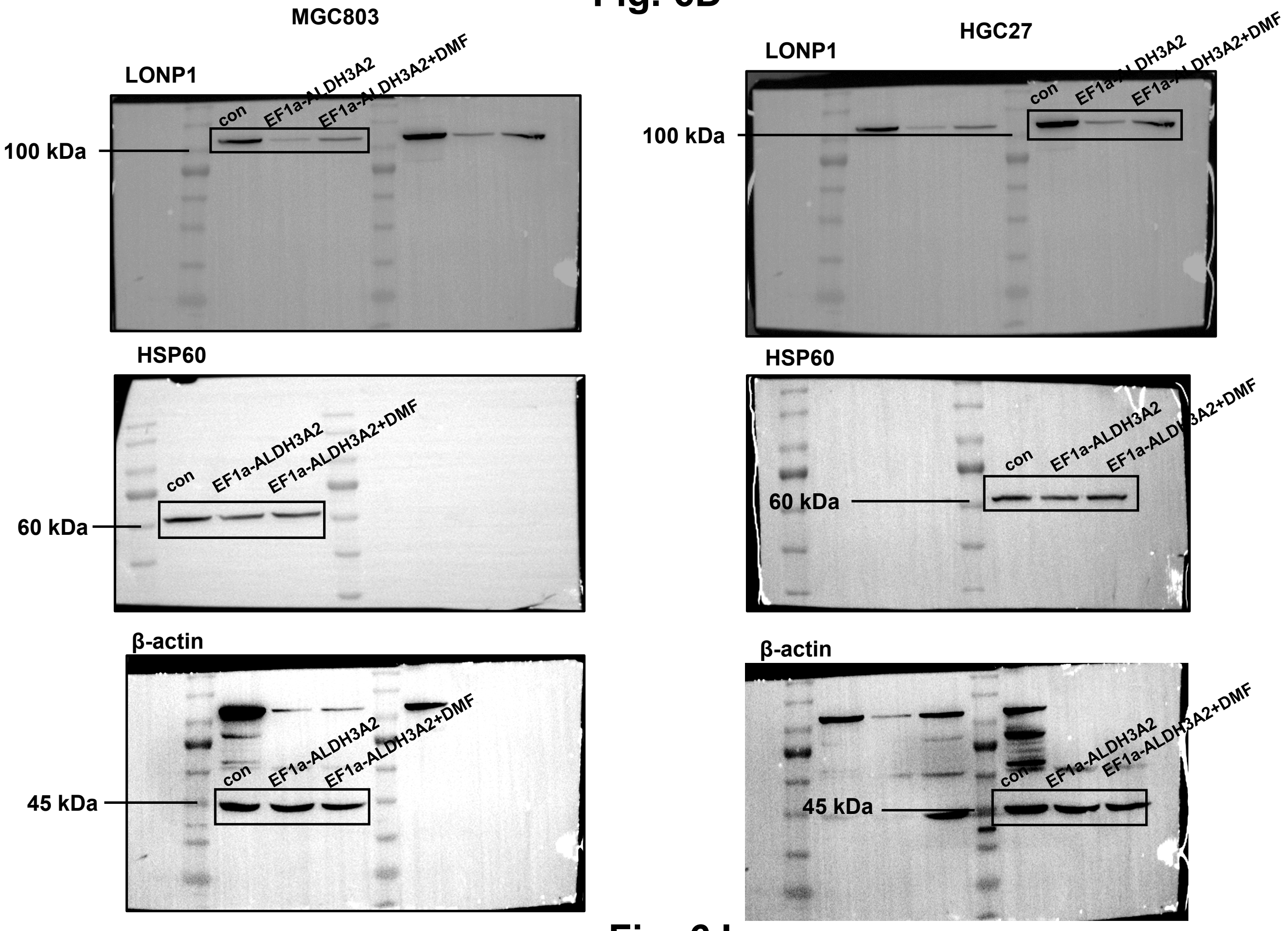
MGC803
HGC27
LONP1
con
EF1a-ALDH3A2
EF1a-ALDH3A2+DMF
100 kDa
HSP60
60 kDa
β-actin
45 kDa

Fig. 6J

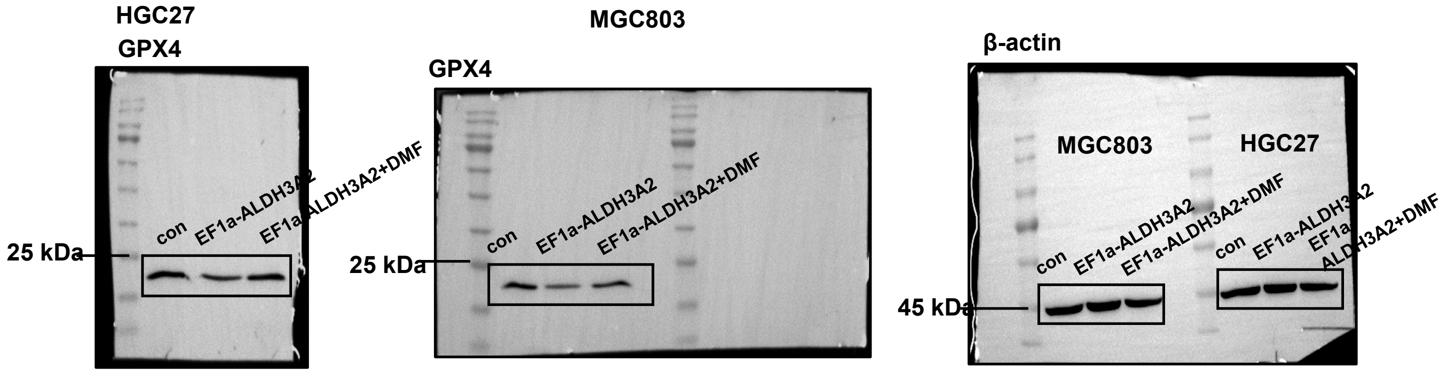

Fig. 6J

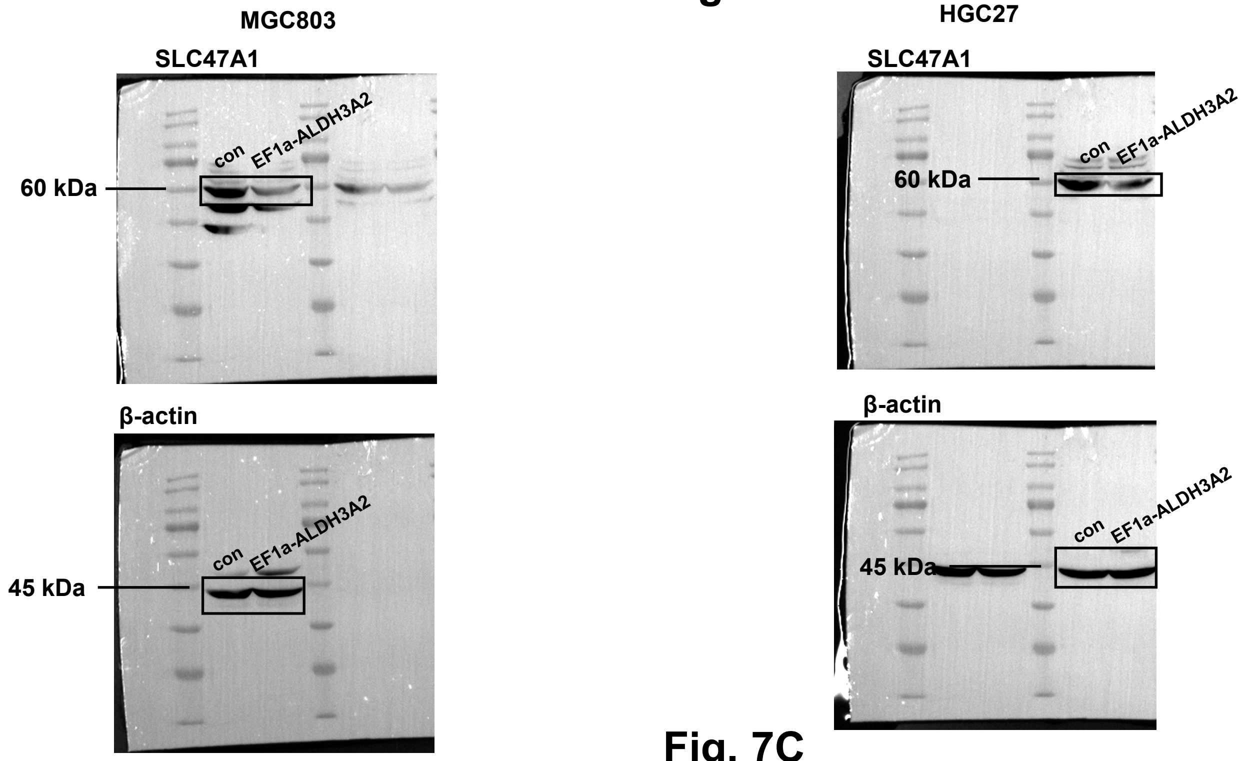

Fig. 7C

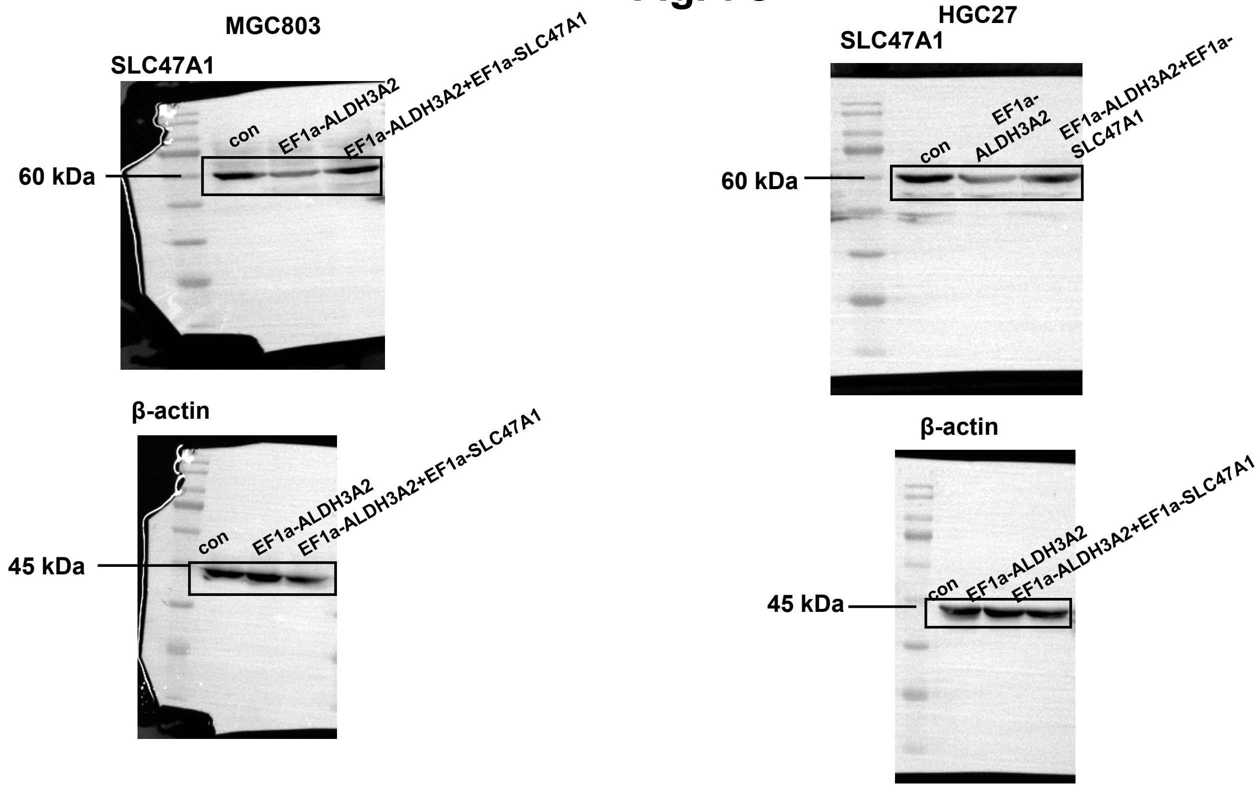

Fig. 7E

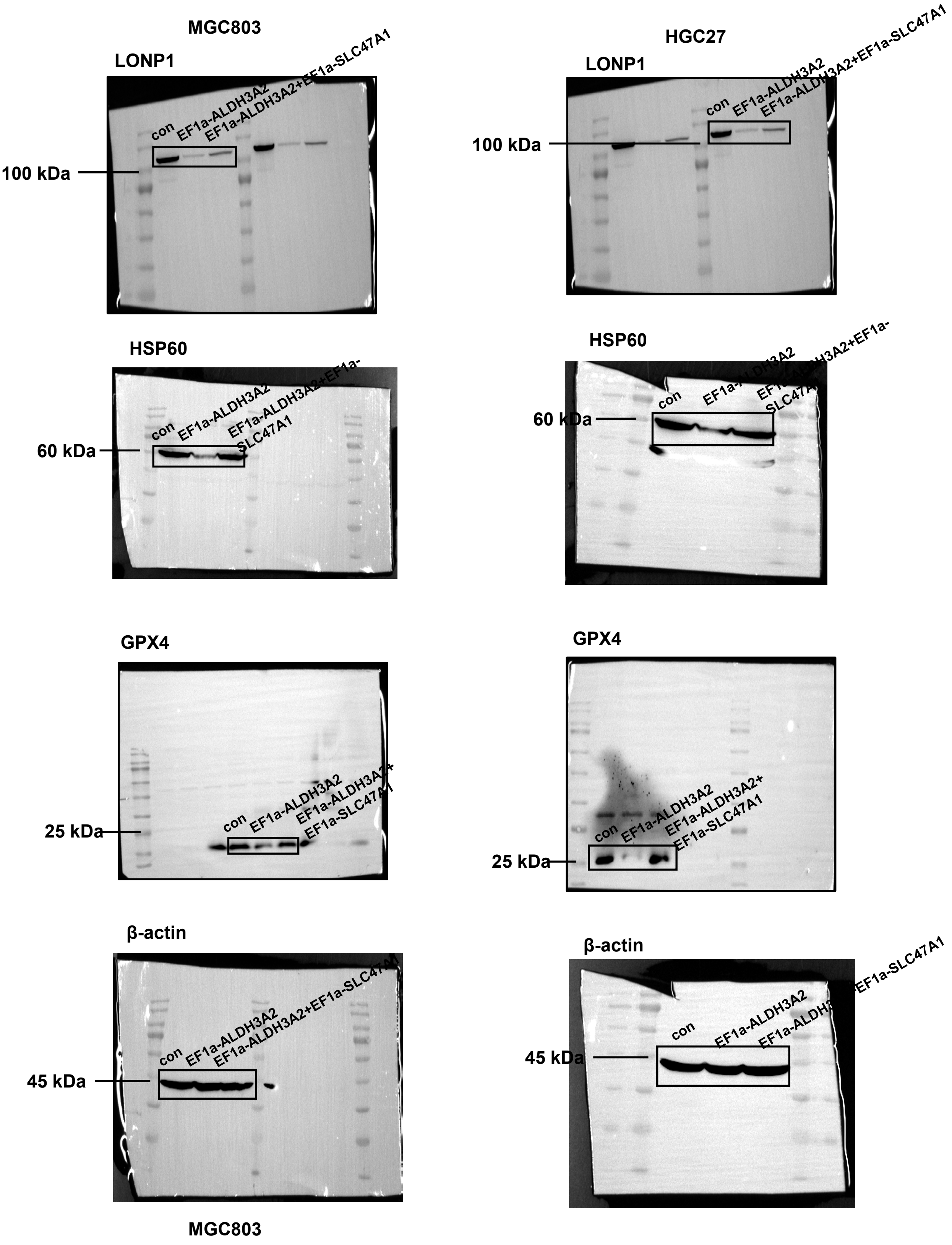
MGC803
LONP1
con
EF1a-ALDH3A2
EF1a-ALDH3A2+EF1a-SLC47A1
100 kDa
HGC27
LONP1
con
EF1a-ALDH3A2
EF1a-ALDH3A2+EF1a-SLC47A1
100 kDa
HSP60
con
EF1a-ALDH3A2
EF1a-ALDH3A2+EF1a-SLC47A1
60 kDa
HSP60
con
60 kDa
GPX4
con
EF1a-ALDH3A2
EF1a-ALDH3A2+
EF1a-SLC47A1
25 kDa
GPX4
con
EF1a-ALDH3A2
EF1a-ALDH3A2+
EF1a-SLC47A1
25 kDa
β-actin
con
EF1a-ALDH3A2
EF1a-ALDH3A2+EF1a-SLC47A1
45 kDa
β-actin
con
EF1a-ALDH3A2
EF1a-SLC47A1
45 kDa
MGC803

Fig. 7I

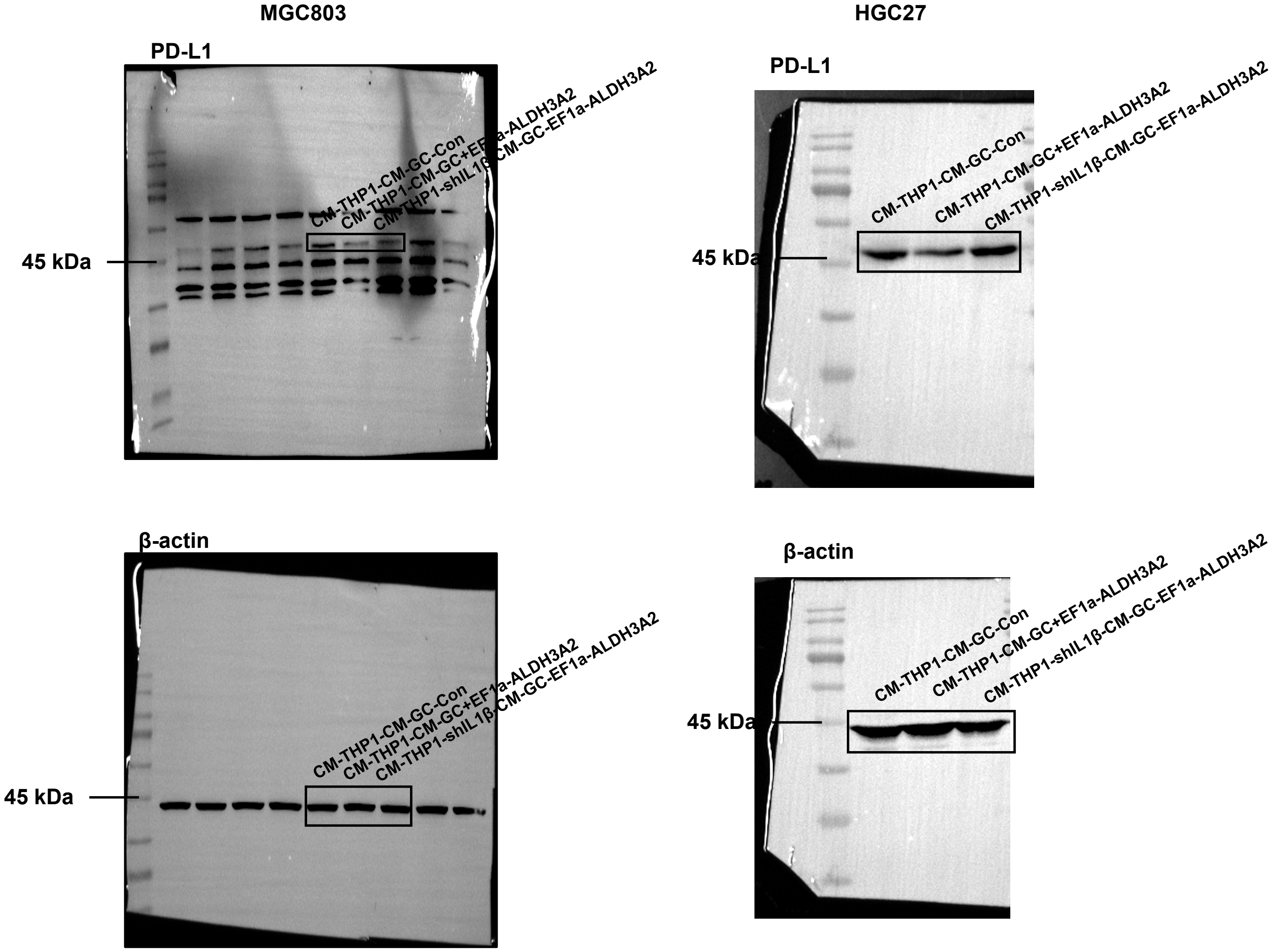

MGC803
HGC27
PD-L1
PD-L1
45 kDa
45 kDa
CM-THP1-CM-GC-Con
CM-THP1-CM-GC+EF1a-ALDH3A2
CM-THP1-shIL1β-CM-GC-EF1a-ALDH3A2
β-actin
β-actin

Fig. 9F

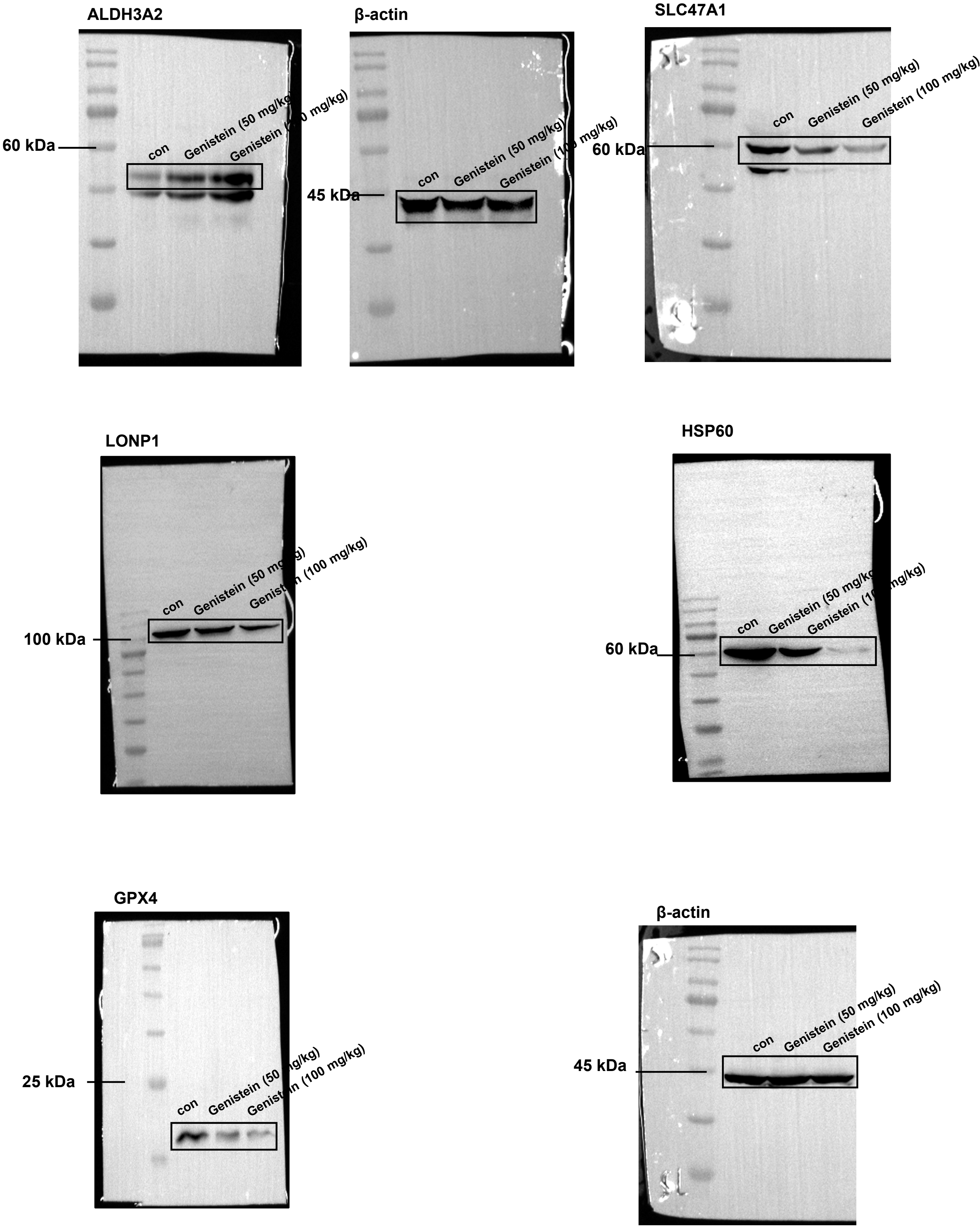
ALDH3A2
60 kDa
con
Genistein (50 mg/kg)
β-actin
45 kDa
con
Genistein (50 mg/kg)
SLC47A1
60 kDa
con
Genistein (50 mg/kg)
Genistein (100 mg/kg)
LONP1
100 kDa
con
Genistein (100 mg/kg)
HSP60
60 kDa
con
GPX4
25 kDa
con
Genistein (100 mg/kg)
β-actin
45 kDa
con
Genistein (50 mg/kg)
Genistein (100 mg/kg)

Fig. 10C

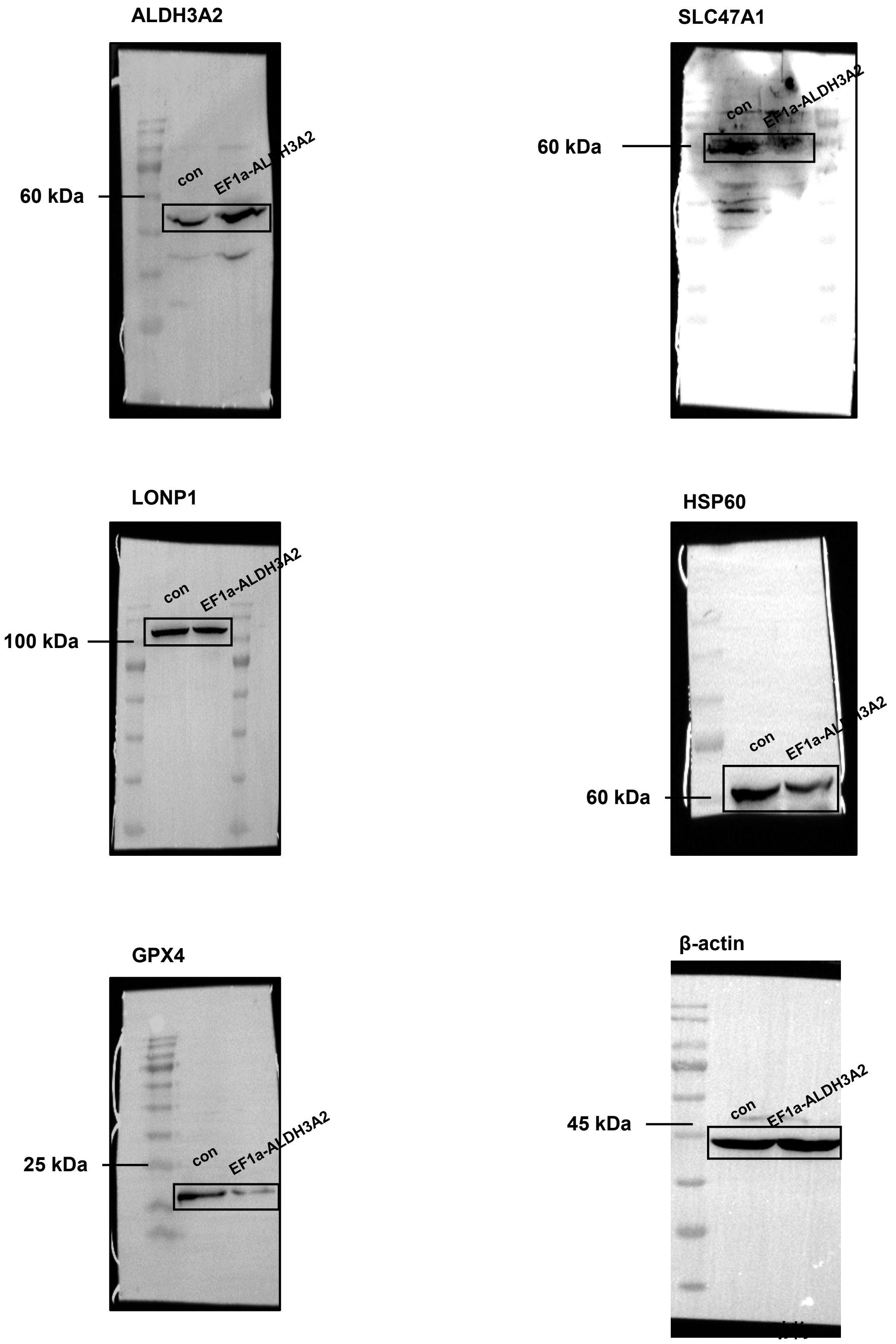
ALDH3A2
60 kDa
con
EF1a-ALDH3A2
SLC47A1
60 kDa
con
EF1a-ALDH3A2
LONP1
100 kDa
con
EF1a-ALDH3A2
HSP60
60 kDa
con
EF1a-ALDH3A2
GPX4
25 kDa
con
EF1a-ALDH3A2
β-actin
45 kDa
con
EF1a-ALDH3A2

Fig. 10G

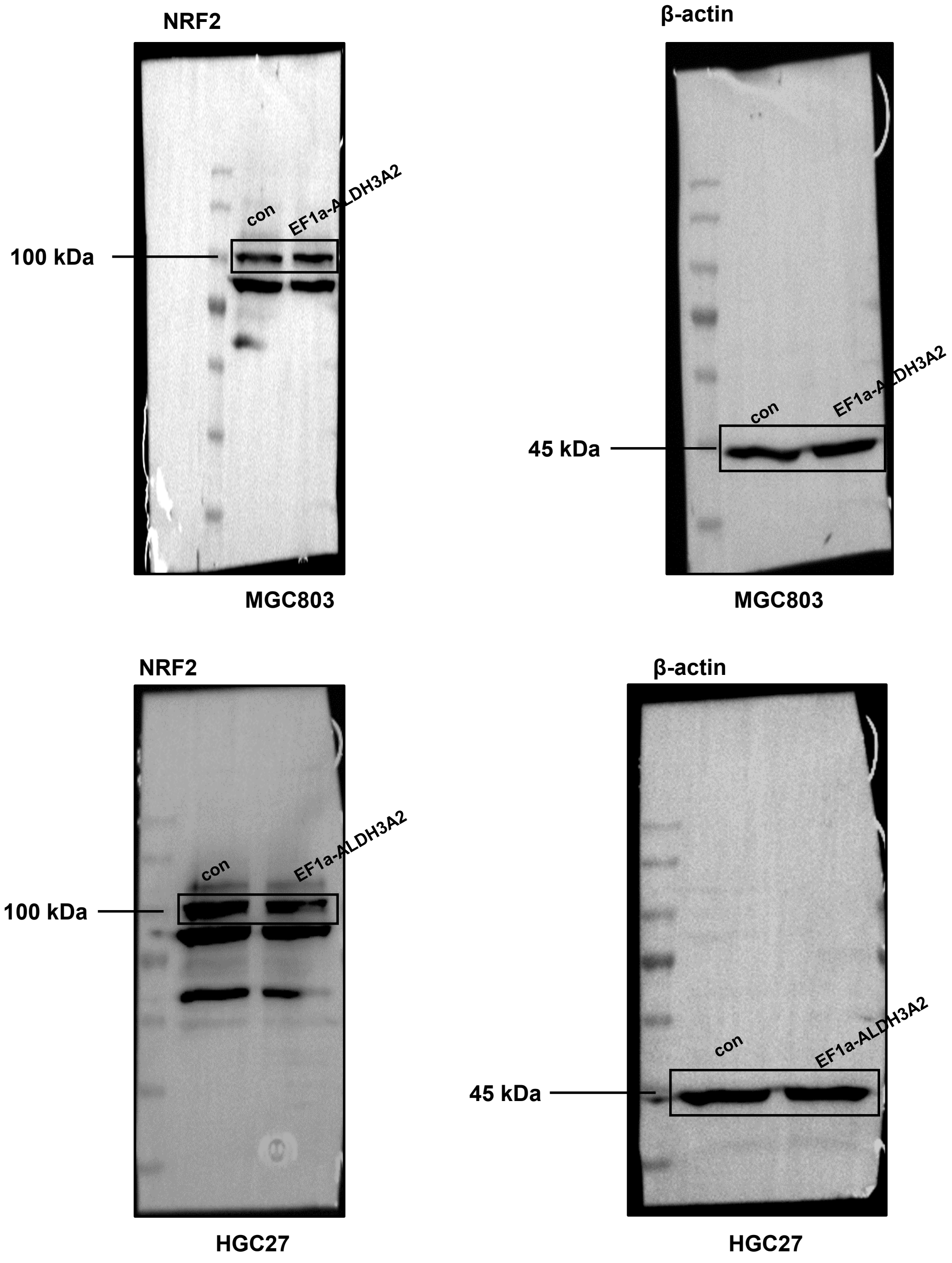
NRF2
con
EF1a-ALDH3A2
100 kDa
MGC803
β-actin
con
EF1a-ALDH3A2
45 kDa
MGC803
NRF2
con
EF1a-ALDH3A2
100 kDa
HGC27
β-actin
con
EF1a-ALDH3A2
45 kDa
HGC27

Fig. S4A-B

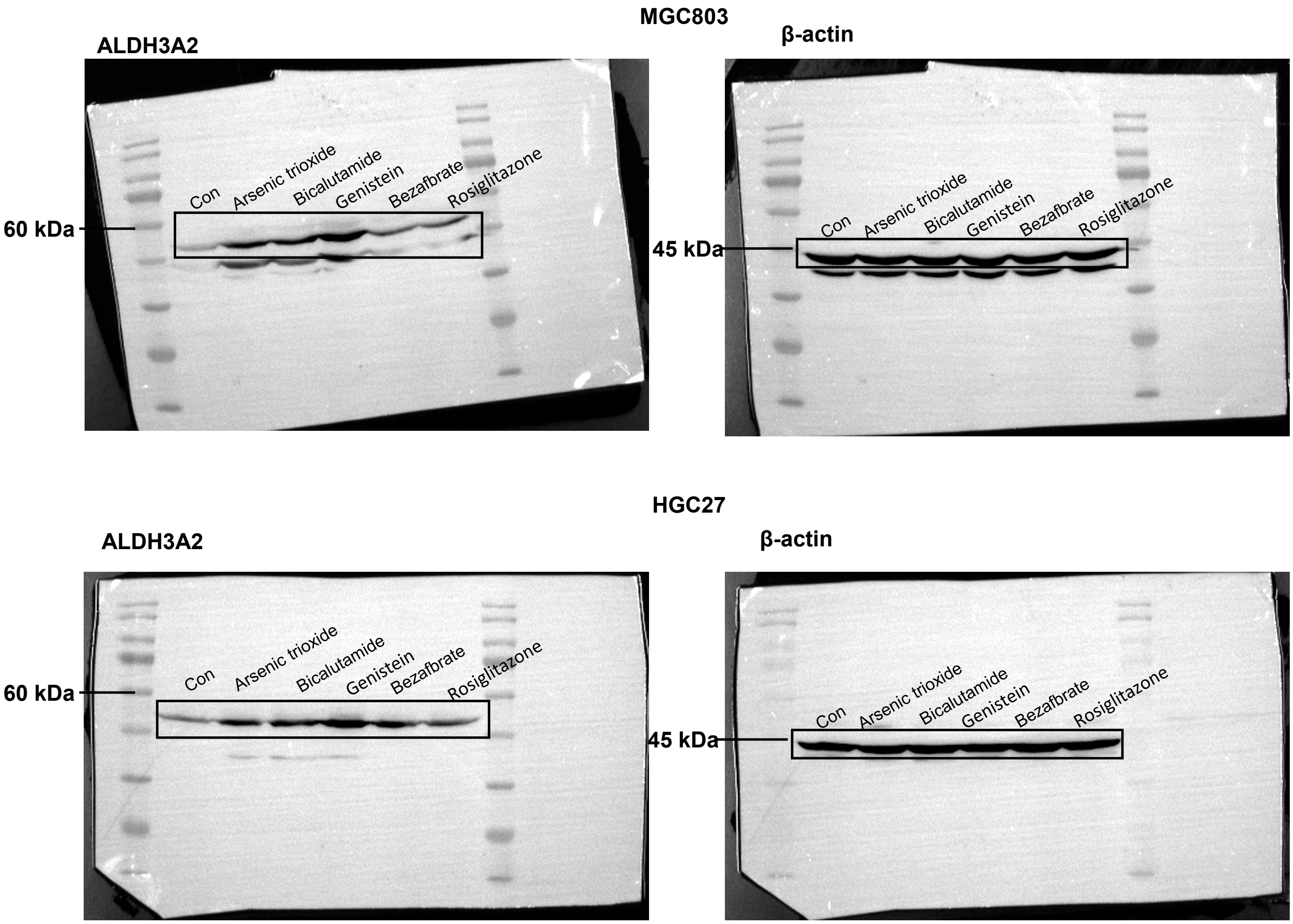
MGC803
ALDH3A2
β-actin
60 kDa
45 kDa
Con
Arsenic trioxide
Bicalutamide
Genistein
Bezafbrate
Rosiglitazone
HGC27
ALDH3A2
β-actin
60 kDa
45 kDa
Con
Arsenic trioxide
Bicalutamide
Genistein
Bezafbrate
Rosiglitazone

Fig. S7C

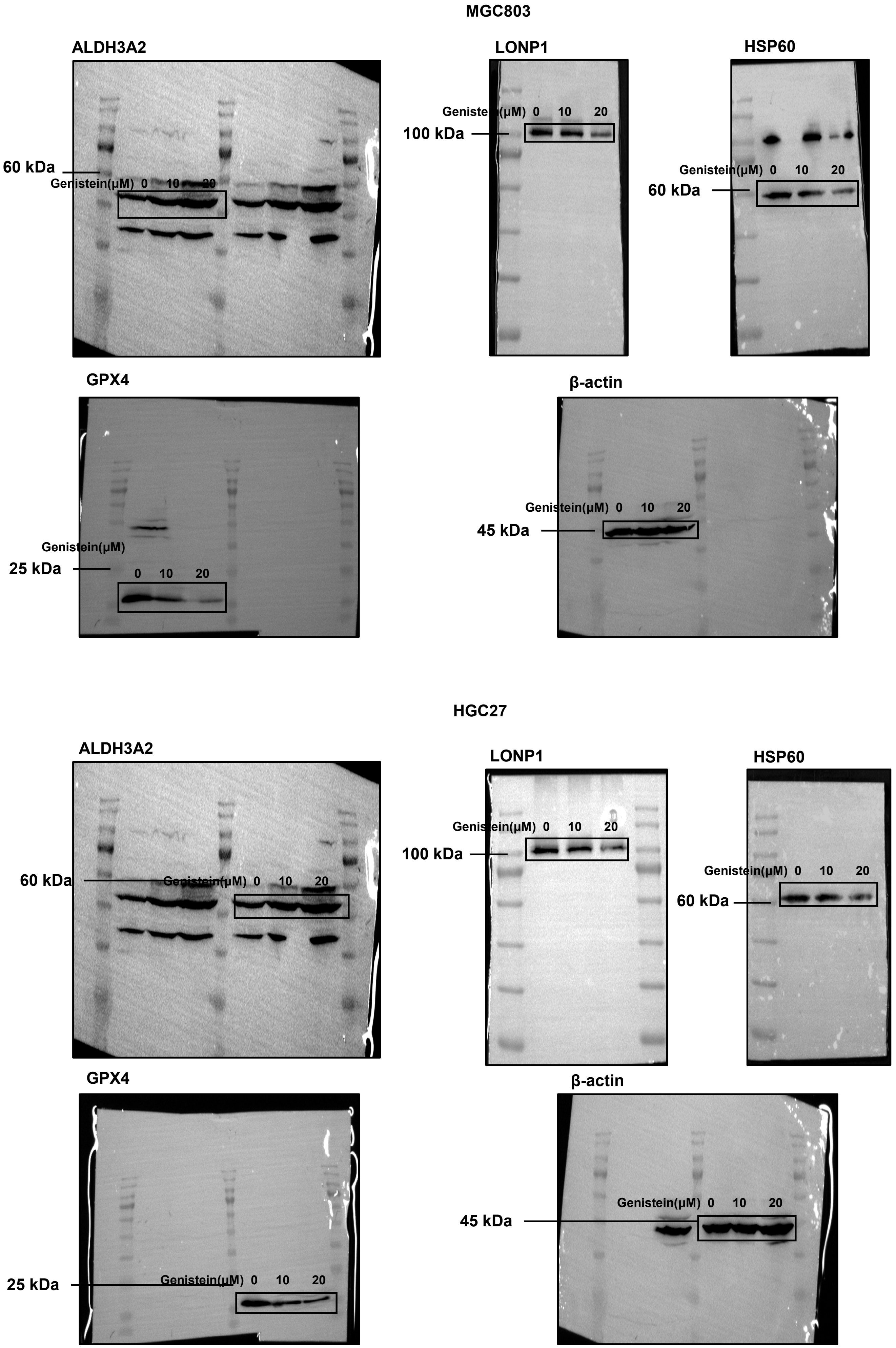
MGC803
ALDH3A2
60 kDa
Genistein(μM) 0 10 20
LONP1
Genistein(μM) 0 10 20
100 kDa
HSP60
Genistein(μM) 0 10 20
60 kDa
GPX4
Genistein(μM)
25 kDa
0 10 20
β-actin
Genistein(μM) 0 10 20
45 kDa
HGC27
ALDH3A2
60 kDa
Genistein(μM) 0 10 20
LONP1
Genistein(μM) 0 10 20
100 kDa
HSP60
Genistein(μM) 0 10 20
60 kDa
GPX4
25 kDa
Genistein(μM) 0 10 20
β-actin
Genistein(μM) 0 10 20
45 kDa

Fig. S7E
